# Evaluation of a Theoretical and Experiential Training Programme for Allied Healthcare Providers to Prescribe Exercise Among Persons with Multiple Sclerosis: A Co-Designed Effectiveness-Implementation Study

**DOI:** 10.3390/jcm14186625

**Published:** 2025-09-19

**Authors:** Yvonne C. Learmonth, Georgios Mavropalias, Kym Wansbrough

**Affiliations:** 1 School of Allied Health (Exercise Science), Murdoch University, Perth, WA 6150, Australia; georgios.mavropalias@gmail.com (G.M.); kym.wansbrough@murdoch.edu.au (K.W.); 2 Personalised Medicine Centre, Murdoch University, Perth, WA 6150, Australia; 3 Centre for Healthy Ageing, Murdoch University, Perth, WA 6150, Australia; 4 Perron Institute for Neurological and Translational Science, Perth, WA, 6009, Australia; 5 School of Medical and Health Sciences, Edith Cowan University, Perth, WA 6027, Australia; 6 Centre for Precision Health, Edith Cowan University, Perth, WA 6027, Australia; 7 School of Psychology, Murdoch University, Perth, WA 6150, Australia

**Keywords:** health education, exercise prescription, allied health, healthcare provider, exercise, behaviour change, continued professional development, implementation

## Abstract

**Background:** Multiple sclerosis (MS) is the most prevalent neurological disorder in young adults, characterised by physical, psychological and cognitive dysfunction. Exercise training is a safe management strategy. Healthcare providers (HCPs) acknowledge deficiencies in awareness, counselling strategies, and resources that prevent them from promoting and prescribing this effective treatment. We implemented an online evidence-based educational programme and evaluated the effect, acceptability, appropriateness, and feasibility of the programme in improving HCP confidence, knowledge, and attitudes towards remote exercise prescription to persons with MS. **Methods**: Physiotherapists and exercise physiologists were recruited and received the educational programme (online theory and 16-week experience of prescribing to persons with MS). Participants’ confidence, knowledge and attitudes towards exercise prescription, as well as their professional quality of life, were our primary outcomes—baseline (T1), immediately post-online theoretical learning (T2), post-application with clients (T3; approximately 16 weeks after T2), and at 12-month follow-up (T4). We gathered participants’ acceptability, appropriateness, and feasibility evaluation at T2, T3 and T4. We analysed the effect on primary outcomes using generalised linear mixed models, with secondary and evaluative outcomes analysed as counts and qualitative themes. **Results**: Of 40 participants who provided baseline data, 24 completed the theoretical programme, and 16 completed the experiential programme. Self-confidence improved significantly (|βs| ≥ 1.27, SEs ≤ 0.31, |zs| ≥ 5.28, ps < 0.001), with large effect sizes (percentage change: 256.8–479.4%). Some theoretical domains framework-based domains have improved, such as beliefs about skills to prescribe evidence-based principles. Participants expressed high satisfaction with the programme and showed increased delivery of implementation behaviour change strategies. **Conclusions**: An online evidence-based education programme for MS care improved HCPs’ self-confidence, perceived skills and delivery of evidence-based exercise behaviour-based prescription.

## 1. Introduction

Multiple sclerosis (MS) is a chronic neurological disease affecting over 33,000 Australians and over 2.8 million people globally [1,2], imposing a significant burden with costs surpassing $2.5 billion annually in Australia alone [2]. Characterised by a broad range of physical and cognitive impairments that can vary widely among individuals, MS significantly affects quality of life. Exercise improves physical impairment and mental health [3,4], making it a critical component of comprehensive MS care. However, despite the known benefits and existence of clinical guidelines, 85% of Australians with MS do not engage in sufficient exercise to achieve meaningful health outcomes [5]. While guidelines for exercise for persons with MS provide comprehensive recommendations [6,7,8], their integration into clinical practice remains limited, particularly in telehealth delivery.

Healthcare providers (HCPs) are often the primary and preferred source of exercise-related guidance for people with MS [9]. However, many HCPs lack the confidence, knowledge, and skills to promote and prescribe exercise effectively within this population [10,11]. Such lack of preparedness to prescribe exercise to patients, including by allied health professions considered experts in doing so (e.g., physiotherapists, occupational therapists and exercise physiologists), is a noted problem within health profession research [12], with professional development opportunity barriers considered a pivotal cause of poor preparedness. Incongruence between consumer need and provider delivery is compounded by challenges in accessing training that addresses the unique needs of persons with MS and the specific barriers they face, such as fatigue, mobility issues, and low mental health [11].

Persons with MS have found remote exercise programmes to be highly acceptable, particularly in rural and remote areas where access to in-person services is often limited [13,14,15,16]. HCP training programmes must equip the healthcare workforce with the confidence, skills and resources to effectively deliver exercise promotion and prescription via telehealth, using evidence-based practices and behaviour change techniques [17]. There is a lack of evaluation of continued professional development courses targeting the HCP workforce [18,19]. Within the evidence for allied HCP education, we know only one past, qualitative study addressing MS. In which community physiotherapists’ use of guidelines following outpatient physiotherapists’ peer support was discussed [20]. Further, there is little education programme evaluation in neurological care more generally [21,22,23,24]. Thus, there is a lack of evidence on which informed decisions can be made by individuals and organisations regarding professional development activities. The need to evaluate online training programmes for HCPs is urgent. Especially given the increasing demand for remote learning activities, which offer time-efficient and accessible solutions [18,19].

In response to this need, we co-designed the Behaviours towards Aerobic and Strength Exercise in MS programme (BASE-HCP) to provide a clinical education to HCPs in the remote delivery of exercise interventions specifically designed for people with MS [25]. The programme followed a self-directed [26], theoretical and experiential framework [27] which emphasised high clarity and high consequence learning principles [24]. The programme was built on established evidence [13,28] and co-designed with three Australian MS physiotherapists and a person with MS, to address HCP education needs for telehealth exercise delivery [11]. Further, before finalising the education programme’s content, we presented the proposed programme to physiotherapists, exercise physiologists and occupational therapists who considered it appropriate for testing in their respective professions. In this study, we aimed to evaluate the effectiveness of the BASE-HCP educational programme in improving HCP confidence, knowledge, and attitudes towards remote exercise delivery for individuals with MS and professional quality of life. In addition, we aimed to establish the acceptability, appropriateness and feasibility of the education programme for clinical usage. In this manuscript, results are restricted to the outcomes of the HCP, which aligns with the first three levels of the Kirkpatrick model to aid the evaluation of education programmes [27], 1. measuring HCPs reaction, 2. knowledge retention and 3. HCP behaviour change.

## 2. Materials and Methods

The study was conducted in accordance with the Declaration of Helsinki and approved by the Institutional Human Ethics Committee (ID 2019/021 & 2023/134). All participants provided informed consent before completing the surveys. We followed the Standards for Reporting Implementation Studies [29].

### 2.1. Study Design

**Figure 1:**
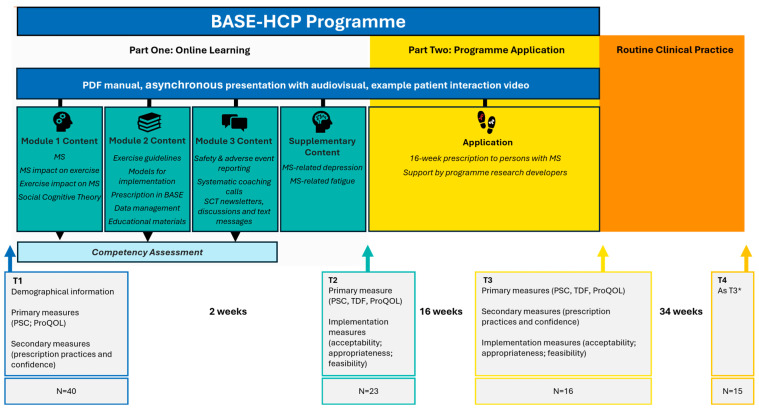
BASE-HCP study design: Intervention components, outcome measures, and participation across the 12-month study. Note. SCT = Social Cognitive Theory; T1 = baseline; T2 = immediately post-online learning; T3 = post-application with clients; T4 = 12-month follow-up. PSC = Practitioner Self-Confidence; TDF = Theoretical Domains Framework; ProQOL = Professional Quality of Life Scale. * Knowledge and Skills not assessed at T4, additional question: “Has the BASE-HCP programme influenced your practice?”.

We completed a one-year longitudinal study to evaluate the self-reported effectiveness of the BASE-HCP educational programme for HCPs (see Figure 1). Participants provided data at four timepoints via the online software Qualtrics (Provo, UT, USA, December 2022): baseline (T1), immediately post-online theoretical learning (T2), post-application with clients (T3; approximately 16 weeks after T2), and at 12-month follow-up (T4). We designed this study to assess changes in HCPs’ confidence, knowledge, and attitudes towards the remote delivery of evidence-based exercise programs for individuals with multiple sclerosis (MS) and implementation outcomes according to Proctor et al.’s framework [30].

### 2.2. Context

The BASE-HCP programme was implemented through allied HCPs within the Australian healthcare system. Programme application occurred between March 2023 and January 2024, coinciding with the Australian government’s expansion of telehealth funding for allied healthcare provision of interventions through Medicare [31], which normalised remote healthcare delivery following COVID-19.

### 2.3. Participant Inclusion Criteria

Physiotherapists, exercise physiologists, and occupational therapists, with interests in neurorehabilitation, were eligible. Inclusion criteria were current practice in healthcare, willingness to participate in the study duration, and availability to complete BASE-HCP training and follow-up assessments. Recruited channels included newsletters and social media of the Australian Physiotherapy Association, Exercise and Sports Science Australia, and Occupational Therapy Australia, as well as emails to clinical managers of allied health clinics across Australia. We excluded participants if they were not one of the above professions or if they were students.

### 2.4. Outcome Measures

We collected demographic and professional information at T1, including age, sex, geographic region, clinical role, primary area of work, caseload of neurological and MS clients, awareness of MS exercise guidelines, and perception of formal training preparation for exercise promotion. We also collected participants’ baseline preferences for the content and conduct of an educational programme to support them in remotely prescribing an exercise programme for persons with MS.

#### 2.4.1. Primary Outcome Measures

The primary outcomes included changes in HCPs’ confidence levels, measured by a modified 8-item Practitioner Self-Confidence Scale (PSC) [32], consisting of three domains: Self-Confidence (3), Attitudes Towards Patients (3), and Natural History and Treatment of MS (2). Lower scores on the PSC indicate higher self-confidence, better attitudes towards patients, and greater knowledge and preparedness regarding the natural history and treatment of MS.

We evaluated knowledge and attitudinal shifts towards exercise promotion using a modified 21-item Theoretical Domains Framework (TDF) [33], capturing changes in seven domains: Knowledge (6), Skills (3), Professional Role (4), Beliefs About Capability (3), Beliefs About Consequences (2), Optimism (2), and Intentions (1). Lower scores indicated more positive responses across all domains. We provide details of the modified PSC and TDF questionnaires in Supplementary Materials S1.1.

HCP professional quality of life (ProQOL) was measured using the 30-item ProQOL Scale [34], consisting of three subscales: Compassion Satisfaction (10), Burnout (10), and Secondary Traumatic Stress (10). Higher scores on the Compassion Satisfaction subscale indicate better outcomes, whereas higher scores on the Burnout and Secondary Traumatic Stress subscales indicate worse outcomes.

The ProQOL and PSC subscales were obtained across all four timepoints (T1 to T4), while the TDF subscales were obtained from T2 to T3 (Knowledge and Skills), or T2 to T4 (remaining domains).

#### 2.4.2. Secondary Outcome Measures

**Figure 2:**
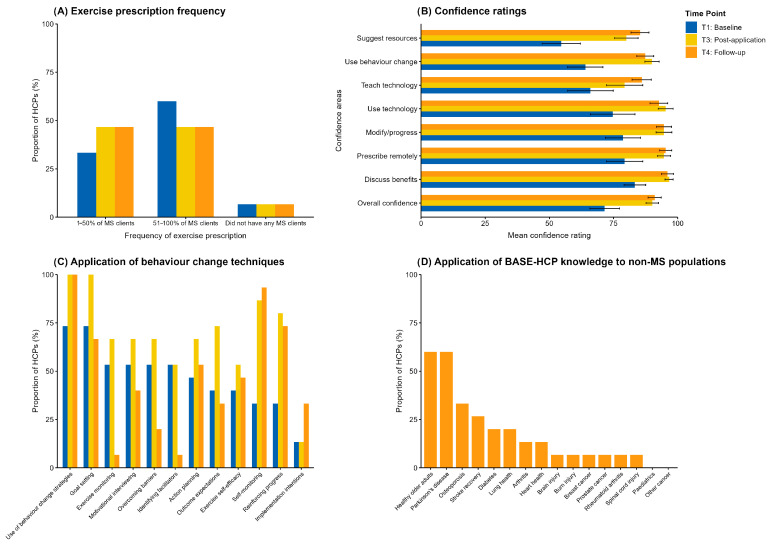
Changes in secondary outcomes of complete-case HCPs (n = 15) from baseline (T1) to post-application (T3) and to follow-up (T4). (**A**) Frequency of exercise prescription across MS clients in routine clinical practice. (**B**) Confidence ratings for remote exercise prescription to MS clients. (**C**) Application of behaviour change techniques in routine clinical practice. (**D**) Application of BASE-HCP knowledge to non-MS clients in routine clinical practice (assessed only at T4). Horizontal bars indicate *M* (SD) data; vertical bars indicate Proportion of HCP (%) data.

Secondary outcomes included frequency of remote exercise prescription to MS clients in routine clinical practice, confidence ratings for remote application components (listed in Figure 2B), and utilisation of behaviour change techniques (listed in Figure 2C). We also assessed post-training application of BASE-HCP principles in routine clinical practice using mixed-methods, incorporating qualitative realist evaluation of knowledge transfer and clinical application beyond the programme delivery period. Participants responded to a binary question regarding whether the BASE-HCP programme influenced their practice. To explore practice changes further, participants responded to qualitative (free text) realist evaluation questions to explore these practice changes further. We provide all questions and assessment items for these secondary outcomes in Supplementary Materials S1.2.

#### 2.4.3. Implementation Evaluation Outcomes

We measured implementation outcomes of acceptability, appropriateness, and feasibility [30]. We assessed acceptability quantitatively through a Likert scale of HCP satisfaction. We assessed appropriateness and feasibility through mixed-methods: quantitative Likert scales and structured qualitative questions with free-text responses. Evaluation of appropriateness involved examining content suitability, time commitment, perceived client appropriateness, perceived client outcomes, applicability to other health conditions, professional delivery considerations, and suggested adaptations. Feasibility assessment focused on participant attrition, time commitment requirements, and barriers to implementation within routine clinical practice. Following realist evaluation principles [35], qualitative responses examined the contexts, mechanisms, and outcomes associated with programme implementation to identify areas for optimisation and inform future scalability.

### 2.5. BASE-HCP Programme

#### 2.5.1. Online Learning

The educational programme comprised online learning modules covering exercise prescription principles for MS, evidence-based content on remote exercise delivery, and behaviour change counselling techniques (Figure 1). We evaluated competency through structured quizzes, which participants completed before being provided with the next module.

#### 2.5.2. Programme Application

The BASE-HCP exercise programme adhered to established MS exercise guidelines [7,8] and consisted of a 16-week structured intervention, delivered to adults with MS who had a Patient Determined Disease Steps [36] score of ≤4 (i.e., mild to moderate disability consistent with the recommendations in the guidelines for exercise in MS) incorporating progressive aerobic and resistance training, delivered four times per week (Figure 1). The intervention systematically incorporated Social Cognitive Theory frameworks [37] throughout, delivered via educational materials, newsletter content, and coaching interactions to enhance exercise behaviour modification in persons with MS. We modified these materials and the content of the online video calls between our HCP participants and persons with MS using co-design principles from existing resources [21,26] and prior research [14,26]. HCP participants received renumeration of AUD50 shopping vouchers per online video call to their clients with MS.

### 2.6. Data Analysis

We prepared and analysed the data using R (version 4.4.2), setting statistical significance to *p* < 0.05. We conducted missing data analysis and visualisation using ‘naniar’ [38] and VIM [39], with Little’s MCAR test performed using the ‘micemd’ package [40]. Missing data analysis revealed dropout as the primary source of missingness (T1 to T2: 42.5%, T2 to T3: 30.4%, T3 to T4: 6.2% dropout), with Little’s MCAR test indicating data were missing at random or missing completely at random [41].

We analysed primary outcomes using the full baseline sample (N = 40). We employed beta generalised linear mixed models (GLMMs) using ‘glmmTMB’ [42] with a complementary log-log link function to accommodate the bounded nature and non-normal distributions of outcome variables [43]. We fit separate GLMMs for each subscale to account for their distinct measurement properties and theoretical constructs. We implemented multiple imputation using predictive mean matching [44] with the ‘mice’ package [45] enhanced with additional tools from ‘miceadds’ [46]. We created 20 imputed datasets given substantial missingness rates (>40% by T4), to reduce bias and maintain statistical power [47]. Models included participant-specific random intercepts, with time as a fixed factor, and two significant predictors identified in preliminary analyses as covariates: (1) T1 awareness of MS exercise guidelines (binary Yes/No response to “Prior to today were you aware of the original exercise guidelines for persons with mild to moderate MS?”); (2) T2 implementation intentions (0–100% scale response to “For how many of the next 10 of your patients with MS do you intend to prescribe remote exercise?”). We pooled results across imputed datasets using Rubin’s rules [48], implemented via ‘mitools’ [49]. For significant findings, we conducted post hoc pairwise comparisons using ‘emmeans’ [50] with Tukey’s adjustment. Effect sizes have been reported as hazard ratios (HRs), with 1.2/0.83, 1.5/0.67, and 2.0/0.5 representing small, moderate, and large effects, respectively. Sensitivity analyses comparing multiple imputation to complete case analysis showed high agreement (93.9%) across parameters, with model diagnostics performed using ‘DHARMa’ (version 0.4 6) [51] and ‘performance’ [52]. Data manipulation and visualisation were performed using ‘tidyverse’ [53]. In Supplementary Materials S1.3, we provide complete methodological details, including missing data analysis, distribution assessment, model selection procedures, and sensitivity analyses.

We analysed secondary outcomes using complete case data, due to their exploratory nature and varying measurement patterns across timepoints. Implementation evaluation employed a stratified approach: learning component evaluation included all participants who completed training modules (i.e., “learning completers”; n = 23), while application component evaluation included only those who completed client implementation (i.e., “application completers”; n = 16), ensuring we captured feedback from all participants with appropriate exposure to each programme component.

## 3. Results

### 3.1. Participant Recruitment and Characteristics

A total of 79 persons visited the study website, with 53 expressing interest in participating. Of these, 40 HCPs (75%) enrolled and completed the T1 (baseline) survey. Study completion rates decreased progressively: 23 participants completed the learning component and T2 survey, 16 completed patient implementation and the T3 survey, and 15 completed the 12-month follow-up T4 survey.

Detailed demographic and professional characteristics are presented in Table 1, with demographic characteristics by timepoint in Supplementary Materials Table S5 Twenty physiotherapists and 20 exercise physiologists participated; however, we were unsuccessful in recruiting occupational therapists. The sample was predominantly female (n = 31, 77.5%), with a mean age of 35.4 years (SD = 9.8), who mainly worked in private practice settings (80.0%). All participants had neurological patients in their caseloads, with 87.5% having recent MS experience. The study included representation from each mainland Australian state, ensuring geographic diversity. Regarding baseline knowledge, 67.5% of HCPs reported prior awareness of MS exercise guidelines. When asked whether formal training had prepared them to promote exercise to clients, 55.0% strongly agreed, 32.5% agreed, and 12.5% were neutral.

### 3.2. Participant Preferences for Education on Remote Exercise Delivery

Via Qualtrics survey, before beginning the programme, we established participants’ baseline preferences for the content and conduct of an educational programme to support them in prescribing an exercise programme for persons with MS. Results are available in Supplementary Materials Figure S1. In doing so, we acknowledge the participants’ likely expectations of the programme. In summary, participants wanted information on various topics and treatment goals. Participants wanted to include behaviour change and exercise prescription in their role when prescribing to clients with MS. Regarding client interaction, our participants mostly wanted their clients to exercise at home, communicate via video call, and have frequent engagement between HCP and client at the start of the programme.

### 3.3. Intervention Effect

#### 3.3.1. Primary Outcomes

We provide complete GLMM results for all subscales in Supplementary Materials Table S6. Here, we present summary statistics: for significant effects, we report the most conservative values (lowest |β|, highest SE, lowest |z|, highest *p*-value) within the significant range, while for non-significant effects, we report the most liberal values (highest |β|, highest SE, highest |z|, lowest *p*-value). Table 2 presents the pooled descriptive statistics and post hoc results for each pairwise time comparison.

##### Practitioner Self-Confidence (PSC)

The PSC indicated a significant change in the domain of Self-Confidence (|βs| ≥ 1.27, SEs ≤ 0.31, |zs| ≥ 5.28, ps < 0.001, |HR| ≤ 0.28, |percentage change| ≥ 71.97%), but not the domains of Attitudes Towards Patients or Natural History and Treatment of MS (|βs| ≤ 0.43, SEs ≤ 0.34, |zs| ≤ 1.77, ps ≥ 0.08). Post hoc pairwise comparisons revealed that, relative to T1, Self-Confidence significantly improved at T2, T3, and T4. However, there were no significant changes in Self-Confidence from T2 onwards.

##### Theoretical Domains Framework (TDF)

For the TDF, we evaluated the domains of Knowledge and Skills at only T2 and T3 (i.e., immediately after theoretical and experiential learning). Significant improvements were observed for both domains (|βs| ≥ 0.97, SEs ≤ 0.29, |zs| ≥ 3.39, ps < 0.001, |HR| ≤ 0.38, |percentage change| ≥ 62.14%). We evaluated the remaining domains at T2, T3, and T4. Beliefs About Consequences significantly changed (|βs| ≥ 0.47, SE ≤ 0.19, |zs| ≥ 3.05, ps ≤ 0.003, |HR| ≤ 1.60, |percentage change| ≥ 60.13%), with post hoc pairwise comparisons revealing a significant improvement at T3 but not T4. We observed no significant changes in the domains of Professional Role, Beliefs About Capabilities, Optimism, or Intentions (|βs| ≤ 0.33, SEs ≤ 0.32, |zs| ≤ 1.13, ps ≥ 0.26).

##### Professional Quality of Life (ProQOL)

For the ProQOL, there was a non-significant trend toward an improvement in Burnout from T1 to T4 (*p* = 0.06). There were no other trends or significant changes in Burnout, Compassion Satisfaction, or Secondary Traumatic Stress over time (|βs| ≤ 0.33, SEs ≤ 0.19, |zs| ≤ 1.68, ps ≥ 0.10). However, the complete case analysis (Supplementary Materials Table S6) identified significant increases in Secondary Traumatic Stress from T1 to T3 and T4.

##### Significant Predictors

T1 awareness of exercise guidelines significantly predicted PSC Self-Confidence, as well as TDF Skills, Professional Role, and Beliefs About Capabilities (|βs| ≥ 0.65, SEs ≤ 0.31, |zs| ≥ 2.08, ps ≤ 0.04). Those who had baseline awareness of exercise guidelines had better scores across these domains. Additionally, both T1 awareness of exercise guidelines and T2 intentions to prescribe exercise) significantly predicted Secondary Traumatic Stress scores (|βs| ≥ 0.01, SE ≤ 0.19, |zs| ≥ 2.47, ps ≤ 0.02). Those with baseline awareness of the guidelines, or who had high post-learning intentions to prescribe, had higher Secondary Traumatic Stress scores.

#### 3.3.2. Secondary Outcomes

##### Changes in Remote Exercise Prescription Practices and Confidence

Indicated in Figure 2A, exercise prescription rates varied, increasing for those with fewer clients with MS, but decreasing for those with more clients with MS. We observed improvements in clinicians’ confidence to deliver remote exercise prescription, increasing from 71.5% at baseline (T1) to 90.1% at T3 and 91.0% at T4. We observed the greatest confidence improvements (Figure 2B) in suggesting remote exercise resources, using behaviour change strategies to encourage remote exercise, and teaching clients how to use devices for remote communication. HCPs’ use of behaviour change strategies also improved (Figure 2C). Self-monitoring, reinforcing progress, and outcome expectations were the most notable increases. However, these changes were not sustained long-term at T4.

##### Post-Training Practice Changes and Knowledge Application

We present our realist evaluation of post-training practice changes and knowledge application in Table 3. In the long term, at T4, 93.3% (n = 14) of participants reported that the BASE-HCP programme had influenced their current practice, noting changes in enhanced evidence-based knowledge, adoption of new clinical techniques, and greater confidence in telehealth delivery and MS management. HCPs demonstrated the programme’s broader clinical utility by applying BASE-HCP knowledge to 14 different patient populations, with healthy older adults, Parkinson’s disease, and osteoporosis being the most commonly targeted conditions (Figure 2D). Transferred programme elements included behaviour change principles, exercise prescription, and patient self-reported exercise, demonstrating the intervention’s applicability beyond MS care.

### 3.4. BASE Implementation Evaluation

#### 3.4.1. Acceptability of the BASE-HCP Programme

**Figure 3:**
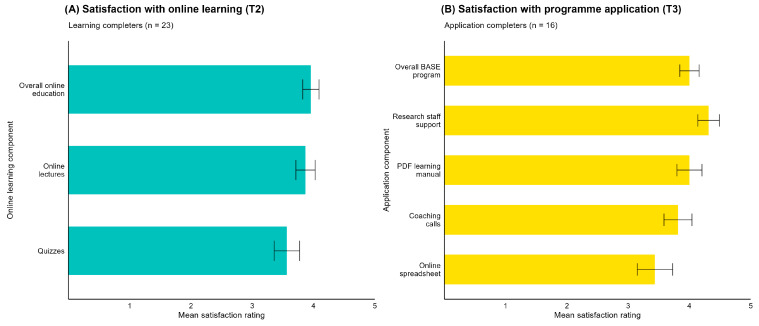
Acceptability of the BASE-HCP programme. (**A**) HCP satisfaction with the online learning component. (**B**) HCP satisfaction with the application component. Bars indicate *M* (SD) data.

HCPs expressed strong satisfaction with the online theoretical learning component (n = 23; mean 4.0/5, 78.3% very/extremely satisfied) and the application component (n = 16; mean 4.0/5, 81.3% very/extremely satisfied; Figure 3).

#### 3.4.2. Appropriateness of the BASE-HCP Programme

##### Appropriateness of Content and Time Commitment

**Figure 4:**
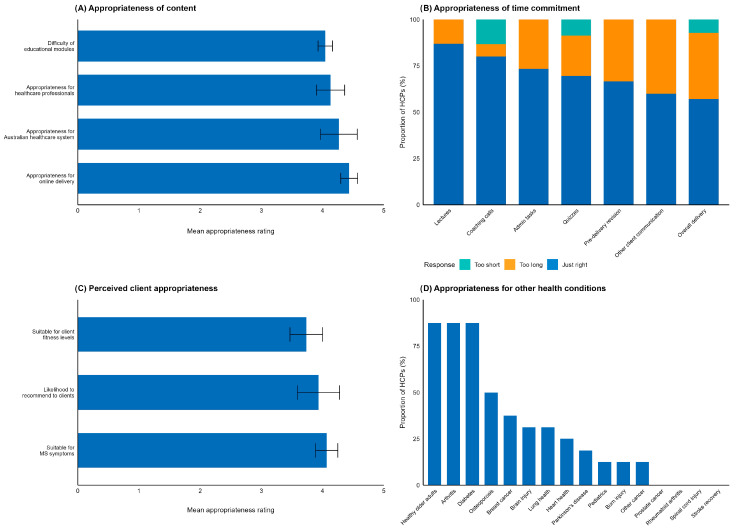
Appropriateness of the BASE-HCP programme. (**A**) Appropriate of content. (**B**) Appropriateness of time commitment. (**C**) Perceived client appropriateness. (**D**) Appropriateness of the programme for application to other health conditions. Horizontal bars indicate *M* (SD) data; vertical bars indicate proportion of HCP (%) data.

Implementation completers reported all content appropriate (Figure 4A), indicated by all mean scores > 4.0/5. Learning completers found the time commitment for online video lectures and assessments acceptable (Figure 4B). Implementation completers had varied perceptions of time requirements, with coaching calls rated most favourably and communications outside coaching calls rated least favourably. Overall, the programme delivery time was considered just right by 50.0% (n = 8) of implementation completers, but too long by 31.3% (n = 5).

##### Perceived Client Appropriateness

HCPs rated the programme highly suitable for their MS clients across multiple dimensions (Figure 4C). The programme was rated as most suitable for MS symptoms (mean 4.1/5), followed by likelihood to recommend to other MS clients (mean 3.9/5) and suitability for client fitness levels (mean 3.7/5). These ratings indicate that HCPs perceived the BASE-HCP programme as well-matched to their clients’ needs and would likely recommend it to other individuals with MS.

##### Perceived Client Outcomes

HCPs prescribed the 16-week exercise behaviour training programme to between two and nine persons with MS each, depending on each HCP’s other commitments. We present our realist evaluation of perceived client outcomes in Supplementary Materials Table S7. HCPs reported that clients experienced functional and psychological benefits from the BASE-HCP program. Clients demonstrated improved physical capacity through increased activity levels and exercise adherence while developing greater self-confidence and motivation toward exercise participation. HCPs attributed these positive outcomes primarily to the program’s structured accountability framework and individualised coaching support, which enabled clients to establish sustainable exercise routines and track meaningful progress. However, HCPs identified that programme success was contingent on client engagement and external circumstances. Clients who struggled with attendance or faced competing priorities were less likely to achieve optimal outcomes, highlighting the importance of commitment and appropriate support structures for programme effectiveness.

##### Appropriateness for Application to Other Health Conditions

Participants rated the BASE learning component as moderately appropriate for delivery to HCPs working with clients with other health conditions (mean 3.7/5). When asked which populations would most benefit from a modified BASE programme (Figure 4D), healthy older adults, arthritis, and diabetes were commonly chosen (each selected by 93.8% of participants), followed by osteoporosis (50.0%) and breast cancer (37.5%).

##### Appropriateness for Professional Delivery

Participants identified specific circumstances where BASE-HCP would be most appropriate for professional delivery (Table 4). Participants recommended the programme for clinicians within the same profession and those seeking telehealth experience and continuing professional development. For clients, participants recommended the programme particularly for remote clients or those who have anxiety about leaving home, for those who demonstrate good digital literacy, or for clients requiring structured exercise support. However, participants did not recommend the programme for clinicians with minimal time or interest.

For clinicians from different professions, participants endorsed a broad range of healthcare providers (e.g., general practitioners, nurses, and other allied health professionals), provided they possessed fatigue and disability awareness and exercise knowledge. Participants did not recommend the programme for clinicians lacking MS experience, passive treatment providers, those working outside their scope of practice, or for unmotivated clinicians. Notably, there was a contradiction regarding novice clinicians. While some participants suggested the programme was appropriate for those new to MS, others indicated it was unsuitable for students or new graduates.

Regarding client characteristics, participants consistently recommended BASE-HCP for geographically remote individuals who have anxiety about leaving home, demonstrate good digital literacy, and follow structured programs well. Conversely, the programme was deemed inappropriate for highly disabled clients with complex needs requiring in-person support, highly active clients with advanced exercise capacity, and newly diagnosed patients needing intensive guidance.

##### Suggested Adaptations for BASE-HCP Implementation

Participants identified numerous adaptations to enhance BASE-HCP implementation across learning and application components (Table 4). Participants recommended structural improvements for the learning component, including mandatory lectures, enhanced case studies and role-play scenarios, and content expansions covering topics such as fatigue management, heat sensitivity, and exercise during relapses. They also suggested broadening the program’s scope to other neurological conditions and adding advanced content for experienced healthcare professionals.

Regarding the application component, participants proposed programme structure modifications including fall risk screening, shorter duration options (12–13 weeks), and incorporating in-person sessions. Exercise content enhancements included expanded variety across difficulty levels and additional MS-specific information. Technology improvements focused on simplifying data entry, automating communications, and developing healthcare provider planner functionality. Additional suggestions included manual improvements (content reduction, hyperlinks, paper diary alternatives) and enhanced support resources such as equipment provision and post-programme referral pathways.

##### Suggested Adaptations for Scaling to Other Health Conditions

Participants identified key adaptations required to scale BASE-HCP to other health conditions (Table 4). Participants emphasised the need for population-specific modules incorporating condition-relevant background information, pathophysiology, and contraindications, alongside enhanced behaviour change coaching components for the learning component.

Application component adaptations focused on exercise prescription modifications and support considerations. Exercise prescription changes included tailoring programs to specific populations, expanding aerobic exercise variety, and incorporating seated exercise options for individuals with low mobility. Participants highlighted the importance of greater individualisation to accommodate diverse condition-specific needs. Support considerations included providing in-person assistance for clients with higher disability levels, evaluating the appropriateness of online delivery modes for varying cognitive abilities, adapting communications to be either generic or disease-specific, and incorporating condition-specific outcome measures to ensure relevant progress monitoring.

#### 3.4.3. Feasibility of the BASE-HCP Programme

##### Participant Attrition

Participant attrition presented a feasibility challenge, with 17 (42.5%) enrolled participants dropping out between baseline and completion of the learning component, 24 (60.0%) by the implementation phase, and 25 (62.5%) by 12-month follow-up. Nine (39.1%) participants did not complete the programme due to lack of time/other work commitments, six (26.1%) provided personal reasons for non-completion, one (4.3%) required more renumeration, one (4.3%) had not received support from their manager and six (26.1%) participants stopped responding to our emails and phone calls.

##### Feasibility of Time Commitment

Completing participants retrospectively reported estimated time commitments for programme components (Table 4). The learning component required a median of 4 h for lecture completion, 5–20 min per quiz, and 30 min for revision during the application phase. For the application component, participants spent a median of 35 min per patient on coaching calls (with initial and final calls extending to approximately 45 min), 20 min on administrative tasks, and 15–30 min on other communications per patient.

Participant responses were mixed when comparing BASE-HCP delivery time to their routine clinical practice. Five participants reported spending more time than usual, attributing this to increased planning requirements and more comprehensive acute care provision. Three participants indicated similar time commitments to standard practice. Six participants reported reduced time investment compared to standard clinical delivery, citing benefits such as shorter assessments, elimination of travel time, and time-saving resources provided by the programme structure.

##### Barriers to Implementation in Routine Clinical Practice

Most participants reported no barriers to implementing the programme in routine clinical practice (Table 4). Among those who identified barriers, three main categories emerged: contextual factors (time commitment, lack of current MS or remote patients, and desire for more exercise prescription autonomy), patient-related barriers (highly disabled or poorly motivated patients), and technology and equipment barriers (equipment requirements/availability and complex technology platforms).

## 4. Discussion

Healthcare providers are the preferred exercise promotion and prescription source for persons with MS [9]. They have reported lacking the confidence, knowledge and skills to effectively promote and prescribe exercise this population [10,11]. We co-designed a training programme [11,25,28] to address the professional development needs among allied HCPs providing care to persons with MS in Australia. Here, for the first time, we test the efficacy of a training programme to improve HCPs’ confidence, knowledge, and attitudes towards exercise promotion in MS, as well as the impact of the programme on their professional quality of life. We also evaluate the programme’s acceptability, appropriateness, and feasibility, while examining whether training translates into measurable changes in clinical practice.

Our training programme resulted in substantial and sustained improvement in self-confidence to prescribe online exercise to persons with MS; our detailed analysis on acceptability and appropriateness explains this result mechanistically. Participants commended our evidence-based theoretical content for addressing knowledge gaps, which we achieved by clearly communicating anticipated outcomes for persons with MS. Our structured experiential programme also provided concrete tools and protocols, which participants praised for giving them the confidence to apply their learning [54]. The experience of delivering the programme, whilst receiving support from experienced peers (i.e., the research team), offered real-world application of their learning, reinforcing competence and aligning with Kolb’s Experiential Learning Model [27]. Further, our study embedded self-reflection through the questions on acceptability and appropriateness, specifically when we asked participants to provide their perceptions on outcomes they and their peers may experience from the training and what outcomes their clients would experience.

A meta-analysis of learning activities to enhance physiotherapist clinical expertise and practice [19] identified mixed results regarding clinical practice change across four blended (in-person and online) learning courses. Our study adds evidence of improved self-confidence, translated into an increase in several clinical competencies, including increased use of behaviour change techniques, greater support for clients using digital health technology, and application of BASE-HCP techniques with their own non-study clients (non-study and non-MS). Within the MS literature, exercise interventions embedding behaviour change techniques are considered more effective than exercise alone [55,56]. Factors including older age, lower socioeconomic status and disease-related impairment are associated with reduced telehealth usage in persons with MS [57,58]. Australians with MS have highlighted the need for support to prepare for and use telehealth [59], and Australian allied healthcare providers have asked for support to prepare their clients to use telehealth [11]. Our programme appears to have met these needs, yet it is still to be established whether the telehealth aspect of our programme is appropriate across all ages, socioeconomic groupings and impairment types in MS. However, HCPs reported adopting skills and application into other clinical populations, including clients with neurological conditions, healthy older adults and clients with other clinical conditions, is a positive sign of programme success beyond across different populations.

However, we note a decline in the long-term use of behaviour change techniques, suggesting a need for mechanisms to identify barriers and ensure intervention components embed long-term use of behaviour change techniques. Relevant mechanisms may include identifying barriers to the use of behaviour change techniques, developing and using intervention components to enhance appropriate choice in behaviour technique, ensuring techniques are based on established theory and providing follow-up training or continuous (peer) support [60,61,62]. Most of our participants worked in private clinics, reducing their access to traditional peer support. Australia has a decade-long history of harnessing clinical networks (often called communities of practice) for sharing healthcare information [63]. Such networks take time to develop, and operate best when focused on local contexts, common goals and condition-specific needs [64]. In this case, the focus on education of HCPs to support exercise promotion and prescription through behaviour change in clinical populations, such as MS. Development of such a clinical practice network may be facilitated by funding to support attracting members, upholding a democratic structure and culture and identifying and supporting key stakeholders [65]. Furthermore, allowing for participation over various types and levels of engagement with ongoing monitoring and evaluation are critical facilitators, which should be tested as a logical next step to promote systemic and long-term use of evidence-based strategies for HCP-led exercise behaviour change in persons with MS [60].

Another explanation of the decline in behaviour change techniques may be explained by the relationship between the patient and HCP. Evidence indicates that a poor or unestablished patient-provider relationship can present a barrier to the use of behaviour change techniques [66]. In our study, we did not gather details on the duration or nature of the relationship between persons with MS and the HCP during the routine clinical practice time-period (prior to timepoint 4), and therefore the relationship with new patients may not have been the same as the relationship with patients during the study period. Future research should examine whether different behaviour change techniques are suitable for various patient groups, considering the duration of care and other demographic and social factors.

Our use of the widely accepted TDF provided strong evidence of participants’ attitudes and beliefs about behaviour. Our programme resulted in participants perceiving improved knowledge and skills. This validates the intended content of the programme to successfully increase knowledge and skills to promote exercise in MS. We consider the experiential component allowed participants to witness the programmes consequences with their clients, reinforcing their beliefs on its likely positive outcome. Our evidence indicates this motivated their change in practice, translating their BASE-HCP knowledge and skills beyond the programme into the care of other clients. With our participants indicating the scope of the programme application in a range of non-clinical (e.g., healthy older adults) and clinical populations (e.g., arthritis and diabetes). Knowledge acquisition, a level two outcome within the Kirkpatrick framework [67], is commonly assessed, and generally improves in comparison with a control group, in studies of allied health (e.g., physiotherapy) learning programmes [68]. Whilst our study informally monitored knowledge, through module quizzes, and did not benefit from including a comparator control group, our reported improvement in perceived knowledge of evidence-based practice likely aided confidence and led to many positive outcomes. A future comparative study of the programme may clarify results further, and mediate for any external factors, such as natural professional development within HCP participants.

Few past studies have monitored physiotherapists’ attitudes and beliefs concerning learning model studies [69,70],, identifying no change in these domains, and we are unaware of any in clinical exercise physiologists [68]. Our results indicated no change in Professional Role, Beliefs About Capabilities, Optimism, or Intentions. This result may be explained by a bias in recruitment to the study, in that the recruited professionals already had high beliefs in each of these areas, with 87% of participants reporting at the start of the study that their formal training had prepared them to promote exercise to clients. Further, our results showed that those who were aware of the MS guidelines and identified high intentions towards prescribing exercise at the start of the programme had associated high baseline scores for Skills, Professional Role, Beliefs About Capabilities, or Intentions.

Similar to past studies of physiotherapy education programmes [21,71], we identified that our training programme showed no discernible change in HCPs’ professional quality of life scores. Our programme’s focus on the potential benefits to the client (with MS) and on the technical skills of delivering behaviour change and exercise progression may explain this result, rather than a focus on the meaning and purpose of why such application of these skills may improve outcomes for the individual healthcare providers and the healthcare system in general. Further, as our intervention focused on one training and development programme to prescribe an intervention to persons with MS, a professional quality of life tool that captures this may be more appropriate. A recently developed and validated Physical Therapists’ Quality of Life tool [72], informed by the ProQOL [34], measures opportunities for training and development programs as one of five domains measuring factors that influence experiences at work. However, these tools and others recommended for measuring workplace innovations [73] may be better used for monitoring job-wide professional quality of life rather than focusing on one training program.

Overall, we identified high acceptability and positive feedback on programme appropriateness for those who completed the theoretical and experiential training. Notably, the components and mechanisms within our programme aligned with participants’ baseline preferences for such a programme. We learned important implementation components which could be considered with further investigation, such as the duration of the overall programme and better preparing the programme to allow for potential patient participation barriers such as symptom fluctuation, motivational downregulation and unexpected events.

Our evaluation of the programme’s feasibility provides essential data on time commitments expected for future delivery of the training program. We did, despite ensuring good communication between research staff and participants and providing a financial incentive for study participation, experience high levels of attrition from the programme, with 42.5% of HCPs leaving the programme before completing the experiential stage. This loss affects the overall validity of our result [74]; however, we addressed this through rigorous missing data procedures, including multiple imputation and comprehensive sensitivity analyses (see Supplementary Materials S1.3). Our sensitivity analyses demonstrated 93.9% agreement between complete case and multiple imputation results, supporting the stability of our findings.

### Strengths and Limitations

Our study carefully involved stakeholders’ needs [10,11] and stakeholder (i.e., physiotherapists and persons with MS) involvement in co-designing our training programme. The training content was built on validated evidence [21,28] and designed following clinical education [27] and relevant clinical practice change [17] frameworks. Further, our evaluation of the programme used validated outcomes and implementation frameworks [30] and aligned with evaluation of education model frameworks [26,27].Our study adds to the limited evidence of clinical education programmes for MS [20] or neurological-focused [21,22,23,24] physiotherapists (with no known evidence in exercise physiology). It furthers the vital agenda of supporting healthcare professionals to promote evidence-based practice to optimise outcomes for persons with MS [17]. Within international healthcare systems, exercise physiology is a younger and less-well-represented profession than physiotherapy, and we believe we are one of the first to evaluate an education programme for exercise physiologists [68].

One limitation is that despite attempts to recruit occupational therapists—a clinical profession that is recommended [8], and, in Australia, accredited to promote and prescribe exercise—we could not attract these professionals to participate. The historical development of roles within healthcare professional education may not have strongly emphasised exercise science within the occupational therapy curriculum [75]. Our poor recruitment highlights that careful consideration will be needed to attract other healthcare professionals (e.g., neurologists and nurses) to see value in receiving training in exercise promotion, and where a co-design approach, including representation from all key stakeholders, will be needed in future research. Our study was completed during the COVID-19 pandemic, which may have influenced results. For example, we experienced high attrition rates, with non-completers citing other commitments (either personal or work-related) as the primary reason for attrition. By gathering information on the time commitments of the study from participants, we will now be able to provide clear information for future studies. We provided some remuneration for participants’ time in delivering the programme to clients; however, funding may be required to expand this remuneration to support the pragmatic rollout of the programme within the Australian healthcare system model.

## 5. Conclusions

Persons with MS want to receive exercise promotion from HCPs. Our education programme for HCPs significantly improved their confidence, knowledge and skills in promoting and prescribing exercise to persons with MS. The theoretical learning programme serves as a potential model on which clinician-led exercise promotion could be supported in MS care with referral to exercise specialists for prescription.

The programme’s evidence-based theoretical content and structured experiential learning were highly praised, leading to increased clinical competencies and positive feedback on its acceptability and appropriateness. However, the study noted high attrition, suggesting that overcoming barriers preventing HCPs from accessing educational training should be considered in future iterations of this or similar work. Finally, the study noted a decline in the long-term use of behaviour change techniques, suggesting the need for continuous support of this effective strategy in MS care.

## Figures and Tables

**Table 1:** Participant demographic and professional characteristics (N = 40).

Characteristic	Result
Age	35.4 ± 9.8
Sex	
Female	31 (77.5%)
Male	9 (22.5%)
Region	
New South Wales	5 (12.5%)
Queensland	13 (32.5%)
South Australia	1 (2.5%)
Tasmania	0 (0.0%)
Victoria	8 (20.0%)
Western Australia	11 (27.5%)
Northern Territory	1 (2.5%)
Australian Capital Territory	1 (2.5%)
Clinical role	
Physiotherapist	20 (50.0%)
Exercise physiologist Occupation Therapist	20 (50.0%) 0 (0.0%)
Primary area of work	
Private clinic	32 (80.0%)
Not for profit	3 (7.5%)
State health authority	2 (5.0%)
Other	3 (7.5%)
Caseload, neurological	
0%	0 (0.0%)
1–50%	19 (47.5%)
51–100%	21 (52.5%)
Caseload, MS	
0%	5 (12.5%)
1–50%	31 (77.5%)
51–100%	4 (10.0%)
Aware of MS exercise guidelines?	
Yes	27 (67.5%)
No	13 (32.5%)
Formal training prepared participants to promote exercise to clients	
Strongly Agree	22 (55.0%)
Agree	13 (32.5%)
Neutral	5 (12.5%)
Disagree	0 (0.0%)
Strongly Disagree	0 (0.0%)

Note. Values are presented as M ± SD for continuous variables and n (%) for categorical variables.

**Table 2:** Pairwise Comparisons for Healthcare Providers’ Self-Confidence, Theoretical Domains Framework, and Professional Quality of Life Following the BASE-HCP Programme (N = 40).

Outcome Measure	T1	T2	T3	T4	Comparison	Result (β (SE), z, *p*)	Effect Size (HR (% Change) [95% CI])	Direction of Change
PSC_SC	7.6	4.9	4.2	4.2	T1 vs. T2	1.27 (0.24), 5.28, < 0.001	3.57 (+256.8%) [2.23, 5.72]	↑ ***
	(2.8)	(1.8)	(1.6)	(1.4)	T1 vs. T3	1.76 (0.25), 6.99, < 0.001	5.79 (+479.4%) [3.54, 9.48]	↑ ***
					T1 vs. T4	1.75 (0.31), 5.67, < 0.001	5.74 (+474.1%) [3.14, 10.50]	↑ ***
					T2 vs. T3	0.48 (0.31), 1.58, 0.11	1.62 (+62.4%) [0.89, 2.97]	← →
					T2 vs. T4	0.48 (0.35), 1.37, 0.17	1.61 (+60.9%) [0.81, 3.18]	← →
					T3 vs. T4	<0.01 (0.30), −0.03, 0.98	0.99 (−0.9%) [0.55, 1.78]	← →
PSC_ATP	5.3	6.1	5.6	5.6	T1 vs. T2	−0.41 (0.23), −1.77, 0.08	0.67 (−33.3%) [0.43, 1.04]	← →
	(1.3)	(1.6)	(1.8)	(1.7)	T1 vs. T3	−0.31 (0.27), −1.13, 0.26	0.73 (−26.6%) [0.43, 1.26]	← →
					T1 vs. T4	−0.17 (0.34), −0.51, 0.61	0.84 (−15.7%) [0.44, 1.63]	← →
					T2 vs. T3	0.10 (0.27), 0.35, 0.73	1.10 (+10.1%) [0.64, 1.88]	← →
					T2 vs. T4	0.23 (0.37), 0.63, 0.53	1.26 (+26.4%) [0.61, 2.63]	← →
					T3 vs. T4	0.14 (0.35), 0.39, 0.70	1.15 (+14.9%) [0.57, 2.30]	← →
PSC_NHT	3.5	3.4	3.0	3.3	T1 vs. T2	<0.01 (0.29), <0.01, 0.100	1.00 (+0.2%) [0.57, 1.77]	← →
	(1.1)	(1.4)	(1.1)	(1.2)	T1 vs. T3	0.43 (0.29), 1.46, 0.14	1.53 (+53.5%) [0.86, 2.72]	← →
					T1 vs. T4	0.18 (0.30), 0.60, 0.55	1.20 (+19.8%) [0.66, 2.16]	← →
					T2 vs. T3	0.43 (0.26), 1.62, 0.10	1.53 (+53.2%) [0.91, 2.57]	← →
					T2 vs. T4	0.18 (0.36), 0.49, 0.62	1.20 (+19.6%) [0.59, 2.44]	← →
					T3 vs. T4	−0.25 (0.38), −0.65, 0.52	0.78 (−21.9%) [0.37, 1.65]	← →
TDF_KNO	NA	1.6	1.2	NA	T2 vs. T3	0.97 (0.29), 3.39, <0.001	2.64 (+164.1%) [1.51, 4.63]	↑ ***
		(0.5)	(0.3)					
TDF_SKI	NA	1.6	1.2	NA	T2 vs. T3	0.98 (0.28), 3.49, <0.001	2.68 (+167.7%) [1.54, 4.65]	↑ ***
		(0.6)	(0.4)					
TDF_PRO	NA	1.8	1.6	1.7	T2 vs. T3	0.33 (0.29), 1.13, 0.26	1.39 (+38.7%) [0.79, 2.44]	← →
		(0.8)	(0.7)	(0.6)	T2 vs. T4	0.16 (0.30), 0.52, 0.60	1.17 (+16.8%) [0.65, 2.09]	← →
					T3 vs. T4	−0.17 (0.33), −0.51, 0.61	0.84 (−15.8%) [0.44, 1.62]	← →
TDF_BELCA	NA	1.7	1.7	1.7	T2 vs. T3	0.13 (0.26), 0.49, 0.63	1.14 (+13.7%) [0.68, 1.91]	← →
		(0.5)	(0.5)	(0.5)	T2 vs. T4	<0.01 (0.27), 0.04, 0.97	1.01 (+1.0%) [0.60, 1.70]	← →
					T3 vs. T4	−0.12 (0.25), −0.47, 0.64	0.89 (−11.2%) [0.54, 1.46]	← →
TDF_BELCO	NA	2.4	2.9	2.8	T2 vs. T3	−0.47 (0.15), −3.05, 0.002	0.62 (−37.5%) [0.46, 0.85]	↑ ***
		(0.4)	(0.5)	(0.4)	T2 vs. T4	−0.34 (0.19), −1.80, 0.07	0.71 (−29.0%) [0.49, 1.03]	← →
					T3 vs. T4	0.13 (0.16), 0.80, 0.42	1.14 (+13.7%) [0.83, 1.56]	← →
TDF_OPT	NA	2.1	2.1	2.1	T2 vs. T3	0.05 (0.29), 0.16, 0.88	1.05 (+4.6%) [0.59, 1.85]	← →
		(0.7)	(0.6)	(0.7)	T2 vs. T4	0.12 (0.32), 0.37, 0.71	1.12 (+12.5%) [0.60, 2.09]	← →
					T3 vs. T4	0.07 (0.32), 0.23, 0.82	1.07 (+7.5%) [0.57, 2.01]	← →
TDF_INT	NA	58.6	64.1	57.0	T2 vs. T3	−0.09 (0.19), −0.48, 0.63	0.91 (−8.9%) [0.62, 1.33]	← →
		(36.0)	(32.9)	(36.1)	T2 vs. T4	0.15 (0.29), 0.54, 0.59	1.17 (+16.6%) [0.66, 2.05]	← →
					T3 vs. T4	0.25 (0.28), 0.89, 0.37	1.28 (+28.0%) [0.74, 2.21]	← →
ProQOL_B	19.1	20.4	20.6	21.6	T1 vs. T2	−0.20 (0.16), −1.29, 0.20	0.82 (−18.1%) [0.60, 1.11]	← →
	(4.2)	(4.8)	(5.1)	(4.6)	T1 vs. T3	−0.29 (0.19), −1.50, 0.13	0.75 (−25.4%) [0.51, 1.09]	← →
					T1 vs. T4	−0.41 (0.21), −1.91, 0.06	0.66 (−33.6%) [0.44, 1.01]	← →
					T2 vs. T3	−0.09 (0.19), −0.49, 0.62	0.91 (−8.9%) [0.63, 1.32]	← →
					T2 vs. T4	−0.21 (0.20), −1.03, 0.31	0.81 (−18.9%) [0.54, 1.21]	← →
					T3 vs. T4	−0.12 (0.23), −0.50, 0.62	0.89 (−11.0%) [0.57, 1.40]	← →
ProQOL_C	43.0 (5.4)	42.6 (5.7)	41.7 (5.9)	41.9 (5.3)	T1 vs. T2	0.04 (0.13), 0.30, 0.76	1.04 (+4.0%) [0.81, 1.34]	← →
					T1 vs. T3	0.17 (0.17), 0.99, 0.32	1.18 (+18.2%) [0.85, 1.64]	← →
					T1 vs. T4	0.18 (0.16), 1.13, 0.26	1.20 (+19.7%) [0.88, 1.63]	← →
					T2 vs. T3	0.13 (0.18), 0.70, 0.49	1.14 (+13.6%) [0.79, 1.63]	← →
					T2 vs. T4	0.14 (0.18), 0.76, 0.45	1.15 (+15.1%) [0.80, 1.65]	← →
					T3 vs. T4	0.01 (0.20), 0.06, 0.95	1.01 (+1.3%) [0.69, 1.49]	← →
ProQOL_STS	16.6 (3.7)	17.5 (4.1)	18.1 (4.5)	17.3 (4.5)	T1 vs. T2	−0.21 (0.18), −1.17, 0.24	0.81 (−18.8%) [0.57, 1.15]	← →
					T1 vs. T3	−0.33 (0.19), −1.68, 0.09	0.72 (−27.8%) [0.49, 1.06]	← →
					T1 vs. T4	−0.28 (0.17), −1.62, 0.10	0.76 (−24.5%) [0.54, 1.06]	← →
					T2 vs. T3	−0.12 (0.21), −0.56, 0.58	0.89 (−11.1%) [0.59, 1.35]	← →
					T2 vs. T4	−0.07 (0.20), −0.36, 0.72	0.93 (−7.0%) [0.63, 1.38]	← →
					T3 vs. T4	0.05 (0.21), 0.21, 0.83	1.05 (+4.6%) [0.69, 1.59]	← →

Note. Data presented as mean (standard deviation) for each outcome. Analysis based on our baseline sample of N = 40 with Beta generalised linear mixed models using multiple imputation for missing data and cloglog link function. Pairwise comparisons used Tukey adjustment for multiple comparisons. T1 = baseline; T2 = immediately post-education; T3 = post-implementation with clients; T4 = 12-month follow-up. PSC = Practitioner Self-Confidence Scale (SC = Self-Confidence, ATP = Attitudes Towards Patients, NHT = Natural History and Treatment of MS; lower scores indicate better outcomes); TDF = Theoretical Domains Framework (KNO = Knowledge, SKI = Skills, PRO = Professional Role, BELCA = Beliefs About Capabilities, BELCO = Beliefs About Consequences, OPT = Optimism, INT = Intentions; lower scores indicate better outcomes). ProQOL = Professional Quality of Life Scale (B = Burnout, C = Compassion Satisfaction, STS = Secondary Traumatic Stress; lower scores are better for Burnout and STS, higher scores are better for Compassion Satisfaction). β (SE), z, *p* = beta coefficient (standard error), z-statistic, *p*-value. HR = Hazard Ratio; HR values around 1.2/0.83, 1.5/0.67, and 2.0/0.5 represent small, moderate, and large effects, respectively. Direction of change: ← → = No change; ↑ = Improvement. Asterisks denote significant change (*** *p* < 0.001).

**Table 3:** Post-BASE-HCP practice changes and knowledge application (n = 15).

Realist Evaluation	Question	Thematic Responses (Number of Participants)	Example Quotes
Outcomes	Explain how BASE-HCP training influenced your current delivery of care/current practice	*Improvements in evidence-based knowledge for practice (8):* Behaviour change principles (3), exercise benefits (1), exercise guidelines (1), telehealth methods (1), MS care (2) *New techniques adopted in practice (10):* Behaviour change techniques (5), telehealth exercise promotion (5) *Enhanced practice confidence (7):* Telehealth exercise promotion (5), MS management (2) (n = 14)	“I have implemented more goal-oriented sessions, improving my education of this population.” “I have thought more about the behaviour change component and placed more time looking into things like participants’ beliefs around exercises, etc, than perhaps I did in the past.” “I feel more confident prescribing and progressing walking programs and resistance exercises over Telehealth. I feel more confident in assessing and managing clients with MS, more broadly.”
	If you applied any of the BASE-HCP knowledge to non-MS patients, what parts or elements of the BASE training do you apply to these clients and how?	Behaviour change principles (7) Exercise prescription (3) Patient self-report of exercise (1) (n = 11)	“I apply the basic principles of behaviour change to facilitate adherence to the exercise program, as well as the exercises themselves and progressions.” “Barriers & Facilitators—educating and recording the client on these principles. Goal Setting—the SMART principle, particularly with the NDIS scheme, and reviewing them regularly.” “The main thing I’ve implemented since the BASE programme is providing a consistent programme for 8–12 weeks with the client’s active tracking of what they are doing. I progressed in the programme when I met with them. It has freed up some time as my clients are more self-sufficient with generalised exercise to maintain their physical well-being, and my physiotherapy sessions can focus more on targeted intervention for challenge areas.”

**Table 4:** BASE-HCP implementation evaluation.

Implementation Construct	Realist Evaluation	Question	Thematic Responses	Example Quotes
Appropriateness				
Professional delivery	Contexts	Under what circumstances would you recommend the BASE program to other clinicians, within the same clinical profession as yourself, to deliver to their MS clients?	Suitable HCPs: new graduates, need telehealth experience, need remote professional development Suitable clients: remote/rural, non-NDIS, those with anxiety leaving the home, those with low exercise motivation, those with good digital literacy, those with general exercise needs who follow structure well	“Any clinician (physio/EP) wanting to improve clinical practice & bridge their evidence-practice gap. The BASE program can have future success with peer-learning and discussions on improving clinical practice, having a follow-on effect in the healthcare system.” “If their clients are remote and not exercising already.”
		Under what circumstances would you NOT recommend the BASE program to other clinicians, within the same clinical profession as yourself, to deliver to their MS clients?	Unsuitable HCPs: students/new graduates, those with minimal time or no interest in the programme Unsuitable clients: highly disabled with complex needs (falls risk, cognitive challenges, high mobility disability) requiring in-person support, highly active with higher exercise capacity	“Clinicians with less than 2 years’ experience (and it can be more difficult to coach/assess/check technique/build rapport online).” “When there is a clinical indication for further assessment that requires in-person review and exercise modification.”
		Under what circumstances would you recommend the BASE program to other clinicians, within a different clinical profession than yourself, to deliver to their MS clients?	Suitable HCPs: those new to MS, those with fatigue and disability awareness, those with exercise knowledge (physiotherapists, exercise physiologists, occupational therapists, GPs, doctors and nurses, allied health assistants, speech pathologists, social workers, and dieticians).	“If I felt they had a suitable knowledge and confidence around MS and exercise, I believe they would be more than capable—it is easy to administer, if they can provide continued information when clients have questions.”
		Under what circumstances would you NOT recommend the BASE program to other clinicians, within a different clinical profession than yourself, to deliver to their MS clients?	Unsuitable HCPs: those with no MS or degenerative condition experience, chiropractors/osteopaths, passive treating HCPs, those working outside their scope of practice, those without exercise experience, those without motivation or time to deliver the program. Unsuitable clients: those with complex needs/higher disability, newly diagnosed clients needing close exercise advice	“If they did not have enough confidence and desire to learn the knowledge, if they didn’t have experience with MS clients or exercise prescription and if they did not know/believe the benefits of exercise for MS.” “Patients who have not first consulted an exercise physiologist or physiotherapist to assess their suitability for the program.”
Suggested adaptations for BASE-HCP	Mechanisms	What changes could be made to the BASE program, which would help you to implement this more easily and widely within your clinical practice?	Learning component Course structure and delivery: make all lectures mandatory, include more case studies and role play scenarios, improve quiz question clarity Content additions and expansion: add topics (fatigue management, heat sensitivity, interval training, navigating relapse and exercise, strategies for exercise regression, therapist project management), expand scope of content to other neurological conditions, add advanced content for more experienced HCPs Application component Program structure modifications: add screening for fall risk, offer shorter program options (12–13 weeks) with deload weeks, include in-person session(s), initial set-up coaching calls Exercise content: include additional MS/exercise information, expand exercise options (including a greater variety of difficulty levels) PDF manual: shorten content, add timetables, add hyperlinks; consider separate manuals for learning vs. application, provide paper diaries as an alternative format option Technology improvements: simplify online spreadsheet data entry, implement auto-populated exercise prescriptions, automate emails/texts, develop HCP planner functionality Support and resources: discuss HCP insurance considerations, provide participant training videos for spreadsheets, provide post-program referral to local resources, and provide HCPs with more equipment	“I found the learning component quite dry. There was a lot of sitting and listening. Something more interesting than watching a recorded PowerPoint.” “The content was good. More education and guidelines on how to manage exercise/program during relapses, when patients have high fatigue or anxiety.” “A few clients encountered sicknesses, injuries (chronic and acute)—often having deload can aid in this and also guide improved long-term muscular strength/health benefits.” “More clarity on parameters regarding variation on exercise progression/ Regression.” “The lectures and content before the implementation were comprehensive and greatly helped during the 16 weeks. I appreciated all the information included in the manuals.” “Too much data entry required by participants. If tracking all data, it would have taken 30+ min per day, which participants didn’t have.” “Understanding the legalities of working in an online setting (e.g., disclaimers, security of data/video platform being used, etc).”
Suggested adaptations for other health conditions	Mechanisms	What would need to change about the BASE training program to make it applicable for delivery in other health conditions?	Learning component: Provide population-specific modules (with changes to background, pathophysiology, and contraindications), and provide behaviour change coaching Application component: Exercise prescription adaptations: tailor exercise prescription to population, provide greater variety in aerobic exercise options, provide seated exercise options for conditions/individuals with low mobility, incorporate individualisation Support considerations: consider unique support needs, make communications (e.g., newsletters) generic or disease-specific, and provide condition-specific outcome measures	“Each health condition has its specific details and degree of variation. I think background information on health conditions and the evidence to support the benefit of exercise specific to that condition is very important.” “In Parkinson’s, probably tailoring some exercises to consider movement size and power.” “For mental health populations such as depression, PTSD, and anxiety, more psychological depth within the behaviour change modules would be needed, with more client coaching calls needed in the program.”
Feasibility				
Time commitment	Outcomes	Estimate how much time you spent completing the learning components of the programme (i.e., lectures, quizzes, and revision)	Lectures: Median = 4 h Quizzes: Median = 5–20 min per quiz Revision for application component: Median = 30 min	NA
		Estimate how much time you spent completing the application components of the programme (per patient)	Coaching calls: Median = 35 min (note: first and last calls were ~45 min) Administrative tasks: Median = 20 min Other communications: Median = 15–30 min	
Barriers to implementation	Mechanisms	Are there currently any barriers that would prevent you from implementing this program for more of your MS clients as part of your routine clinical practice?	The majority reported no barriers. Remaining barriers included: contextual factors (time commitment, no current MS patients, no remote patients, and the desire for more exercise prescription autonomy); patient-related barriers (highly disabled patients, poorly motivated patients); and technology & equipment barriers (equipment requirements/availability, complex/differing technology platforms)	“I would automate reminder messages and newsletter emails to save time.” “If my client were highly disabled or had frequent flareups or episodes of poor mobility /health, I would not see this as a good option for them. Face-to-face would be a much better way to determine their capacity/level to exercise.”

Note. NA = Not applicable.

## Data Availability

All available data is provided in the manuscript or Supplementary Materials.

## Supplementary Materials

The following supporting information can be downloaded at: https://www.mdpi.com/article/10.3390/jcm14186625/s1, Table S1. Modified Practitioner Self-Confidence Scale (PSC) Items (presented in order of administration); Table S2. Modified Theoretical Domains Framework (TDF) Items (presented by subscale); Table S3. Self-Developed Secondary Outcome Measures; Table S4. Sensitivity Analysis: Comparison of Multiple Imputation versus Complete Case Analysis Results for Key Model Parameters; Table S5. Participant demographic and professional characteristics at each time point; Figure S1. Participants baseline preferences for an education programme to support remote exercise delivery to persons with MS. (a) Exercise topics of interest for MS client education. (b) Common treatment goals for MS clients. (c) Preferred delivery roles. (d) Preferred exercise environment. (e) Preferred communication methods. (f) Preferred communication frequency. (g) Preferred adherence monitoring methods; Table S6. Generalised Linear Mixed Model Results for Practitioner Self-Confidence, Theoretical Domains Framework, and Professional Quality of Life Following the BASE-HCP Programme (N = 40); Table S7. Perceived client outcomes (n = 15). Associated methodological references in the Supplementary Materials are [32,33,41,76,77,78,79,80,81,82,83,84,85,86,87,88].

## Abbreviations

The following abbreviations are used in this manuscript:

MS	Multiple Sclerosis
HCP	Healthcare professionals
BASE-HCP	Education Programme for healthcare professionals, Changing Behaviour towards aerobic and Strength Exercise
PSC	Practitioner Self-confidence Scale
SCT	Social Cognitive Theory
ProQOL	Professional Quality of Life Scale
TDF	Theoretical Domains Framework
T1	Baseline data collection
T2	Post-theoretical online learning data collection
T3	Post-experiential/programme application data collection
T4	Six to eight months post learning data collection
COVID-19	Coronavirus pandemic 2020–2023
BASE-HCP	Changing Behaviour towards Aerobic and Strength Exercise Programmes delivered to persons with MS
