# Supplementary material for: Evaluation of a Theoretical and Experiential Training Programme for Allied Healthcare Providers to Prescribe Exercise Among Persons with Multiple Sclerosis: A Co-Designed Effectiveness-Implementation Study
Source: J Clin Med. 2025 Sep 19;14(18):6625. doi: 10.3390/jcm14186625 (PMC12470950; doi:10.3390/jcm14186625)
Supplement: Supplementary file 1 [file jcm-14-06625-s001.zip › jcm-3839704-supplementary.pdf]

Journal of
Clinical Medicine

MDPI

Supplementary Materials S1. Supplementary methods

Supplementary S1.1. Primary outcome measures: Modified questionnaires

S1.1.1. Modified Practitioner Self-Confidence scale (PSC)

The PSC scale[1] was originally developed to assess practitioner self-confidence in treating acute low back pain. For this study, items were modified to be MS-specific by replacing references to "low back pain" or general patient populations with "patients with MS" or "persons with MS," while maintaining the original three-factor structure and response scale. The PSC scale (Table S1) uses summed scoring within each subscale. First, items 3, 4, 5, and 6 are reverse scored (1→5, 2→4, 3→3, 4→2, 5→1) to ensure consistent directionality across all items. Subscale scores are then calculated by summing the relevant items: Self-Confidence (items 1, 2, 8; range 3-15), Attitudes Toward Patients (items 3, 5, 6 after reverse scoring; range 3-15), and Natural History & Treatment (items 4, 7 with item 4 reverse scored; range 2-10). Lower summed scores indicate better outcomes, with lower Self-Confidence scores reflecting higher practitioner confidence in exercise prescription for MS patients, lower Attitudes Toward Patients scores indicating more positive attitudes toward MS patients, and lower Natural History & Treatment scores representing greater knowledge and preparedness regarding the natural history and treatment of MS.

Table S1. Modified Practitioner Self-Confidence Scale (PSC) Items (presented in order of administration)

Item #	Item text	Subscale
1	I know exactly what to do to effectively prescribe exercise therapy in patients with MS	Self-Confidence
2	I am very comfortable prescribing exercise therapy in patients with MS	Self-Confidence
3*	There is nothing physically wrong with many patients with MS who complain of not being able to exercise	Attitudes Toward Patients
4*	There is little I can do to encourage patients who do not exercise from developing exercise behaviours	Natural History & Treatment
5*	Patients with MS often have unrealistic expectations about what clinicians can do for them	Attitudes Toward Patients
6*	I often have negative feelings about dealing with patients who have MS	Attitudes Toward Patients
7	Most of my patients with MS are very satisfied with my care	Natural History & Treatment
8	I am well prepared to prescribe exercise to persons with MS now	Self-Confidence

Note. Items adapted from the original PSC scale for low back pain[1] to be MS-specific. Response Scale: 1 = Strongly Disagree, 2 = Disagree, 3 = Neutral, 4 = Agree, 5 = Strongly Agree. Asterisks denote items that are reverse-scored.

S1.1.2. Modified Theoretical Domains Framework (TDF) questionnaire

The TDF questionnaire[2] was originally developed to assess implementation-related constructs across multiple behavioural domains. For this study, items were modified to be specific to remote exercise prescription for persons with MS by replacing generic

implementation references with "prescribing remote exercise to persons with MS" or "remotely deliver exercise using the BASE programme to persons with MS," while maintaining the original theoretical framework and domain structure. The TDF questionnaire (Table S2) uses domain-specific scoring with items rated on a 5-point Likert scale (1 = Strongly Agree to 5 = Strongly Disagree). Domain scores are calculated by taking the mean of items within each domain: Knowledge (6 items), Skills (3 items), Professional Role (4 items), Beliefs About Capabilities (3 items), Beliefs About Consequences (2 items), Optimism (2 items), and Intentions (1 item with 0-100% scale). Lower scores indicate stronger agreement, with lower Knowledge scores reflecting greater familiarity with BASE program content and remote exercise delivery, lower Skills scores indicating higher perceived competency, lower Professional Role scores representing stronger role identification with remote exercise prescription, lower Beliefs About Capabilities scores reflecting greater confidence in prescribing under challenging conditions, lower Beliefs About Consequences scores indicating stronger beliefs in positive outcomes, lower Optimism scores reflecting greater optimism about remote exercise prescription, and higher Intentions scores representing greater intention to prescribe remote exercise to future MS patients.

Table S2. Modified Theoretical Domains Framework (TDF) Items (presented by subscale)

Subscale	Item #	Item text
Knowledge	1	I am aware of the content and objective of the BASE program
	2	I know the content and objectives of the BASE program
	3	I am familiar with the content and objectives of the BASE program
	4	I am aware of how to remotely deliver exercise programs to persons with MS
	5	I know how to remotely deliver exercise using the BASE programme to persons with MS
	6	I am familiar with how to remotely deliver exercise using the BASE programme to persons with MS
Skills	1	I have been trained how to remotely deliver exercise using the BASE programme to persons with MS
	2	I have the proficiency to deliver exercise using the BASE programme to persons with MS
	3	I have the skills to deliver exercise using the BASE programme to persons with MS
Professional Role	1	Prescribing remote exercise to persons with MS is part of my work as a clinician/therapist
	2	As a clinician/therapist, it is my job to prescribe remote exercise to persons with MS
	3	It is my responsibility as a clinician/therapist to prescribe remote exercise to persons with MS
	4	Prescribing remote exercise to persons with MS is consistent with my profession
Beliefs About Capabilities	1	I am confident that I can prescribe remote exercise to persons with MS, when the person with MS is not motivated
	2	I am confident that I can prescribe remote exercise to persons with MS, when there is little time
	3	I am confident that if I wanted to, I could prescribe remote exercise to persons with MS

Beliefs About Consequences	1	If I prescribe remote exercise to persons with MS, it will benefit public health
	2	If I prescribe remote exercise to persons with MS it will have disadvantages for my relationship with persons with MS
Optimism	1	With regards to prescribing remote exercise to persons with MS in uncertain times, I usually expect the best
	2	With regards to prescribing remote exercise to persons with MS I'm always optimistic about the future
Intentions	1	For how many of the next 10 of your patients with MS do you intend to prescribe remote exercise

Note. Items adapted from the original TDF questionnaire for implementation research[2] to be specific to remote exercise prescription for MS. Items are presented grouped by theoretical domain. Response Scale for all Likert items: 1 = Strongly Agree, 2 = Agree, 3 = Neutral, 4 = Disagree, 5 = Strongly Disagree. The Intentions item uses a 0-100% scale.

Supplementary S1.2. Secondary outcome measures: Assessment items

Secondary outcomes were assessed using study-specific measures developed to evaluate clinical practice behaviours, confidence levels, and knowledge application related to remote exercise prescription for persons with MS. Table S3 provides the complete assessment items and response formats for all secondary outcome measures.

Table S3. Self-Developed Secondary Outcome Measures

Construct	Question(s)	Response format	Response options
Frequency of remote exercise prescription in routine clinical practice	If you actively work with clients with MS, what percentage of your MS clients have you prescribed exercise to within the past year?	Single-select categorical	1-50%;<br>51-100%;<br>None;<br>I don't work with MS clients
Confidence ratings for remote application components	How do you rate your confidence to...<br>(1) Discuss the benefits of exercise with your clients?<br>(2) Prescribe home (i.e. remotely delivered) exercise to your clients?<br>(3) Modify, progress or regress a clients remote exercise program<br>(4) Suggest remote exercise resources, programs, and activities, to further educate your clients?<br>(5) Use behaviour change strategies to encourage remote exercise<br>(6) Use a computer, phone etc to communicate with your clients remotely<br>(7) Teach your client to use a computer, phone etc to communicate with you remotely	Rating scale	0-100% (10% intervals)
Utilisation of behaviour change techniques	(1) Do you currently use behaviour change strategies to ensure your clients complete their current rehabilitative exercises at home (e.g. self monitoring, motivational interviewing, goal-setting)?<br>(2) Which behaviour change strategies do you typically employ?	(1) Single-select;<br>(2) Multiple-select	(1) Yes;<br>No<br>(2) Action planning;<br>Implementation intentions;<br>Self-monitoring;<br>Reinforcing progress;<br>Motivational interviewing;<br>Goal setting;<br>Outcome expectations (e.g., long term exercise benefits);<br>Exercise self-efficacy;

			Exercise monitoring; Overcoming barriers; Identifying facilitators (e.g., things which make exercise easier); Others (please type what these are below)
Post-training practice changes and knowledge application	(1) Has the BASE-HCP training influenced your current delivery of care/current practice? (2) If you answered yes in the previous question, please write below how BASE-HCP influenced your current delivery of care/current practice (3) Have you applied any of the BASE-HCP knowledge to any of your non-MS patients? If yes which of these conditions have you applied the BASE-HCP knowledge? (4) If you applied any of the BASE-HCP knowledge to any non-MS patients, what parts or elements of the BASE training to you apply to these clients and how?	(1) Single-select; (2) Free text; (3) Multiple-select; (4) Free text	(1) Yes; No; I don't know (3) Healthy older adults; Paediatrics; Arthritis; Brain injury; Burn injury; Breast cancer; Diabetes; Heart health; Lung health; Osteoporosis; Parkinson's disease; Prostate cancer; Other cancer; Rheumatoid arthritis; Spinal cord injury; Stroke recovery; Other population

Note. Secondary outcome measures were developed specifically for this study. Confidence ratings scored as the average response across the 7 items.

Supplementary S1.3. Detailed data analysis procedure

S1.3.1. Missing data analysis

Missing data analysis revealed participant dropout as the source of missing values, with missingness consistent across all outcome scales. Specifically, there was a 42.5% dropout between T1 (baseline) and T2 (post-education), 30.4% dropout between T2 and T3 (post-implementation), and 6.2% dropout between T3 and T4 (1 year follow-up). Little's Missing Completely at Random (MCAR) test was performed separately for all outcome measures (PSC p = .754, TDF p = .434, ProQOL p = .811), which indicated either MCAR or Missing at Random (MAR) mechanisms[3].

To determine the missing data mechanism and identify auxiliary variables for inclusion in imputation models, we examined potential predictors of missingness by comparing participants with and without missing data across several variables[4]. Categorical variables were analysed using chi-square tests (or Fisher's exact tests when cell counts were small), including: sex, healthcare profession, experience with neurological cases, experience with MS cases, awareness of exercise recommendations, preparedness for exercise prescription, and prior experience prescribing exercise. Several continuous variables were also compared using independent samples t-tests, including: baseline average confidence and all outcome measures from previous timepoints. Two significant predictors were identified: 1) T1 awareness of MS exercise guidelines (binary Yes/No response to "Prior to today were you aware of the original exercise guidelines for persons with mild to moderate MS?"; χ^2 = 4.27, p = .04); 2) T2 implementation intentions (0-100% scale response to "For how many of the next 10 of your patients with MS do you intend to prescribe remote exercise?"; t = -2.81, p = .011). The presence of significant predictors indicated that data were likely MAR, supporting the appropriateness of multiple imputation[5]. However, a power analysis for these predictor analyses revealed limited statistical power (32-47% power to detect medium effects), suggesting the predictor analyses were underpowered.

Consequently, we considered two approaches to multiple imputation: a complex model incorporating all potential predictors versus a simplified model using only the two significant predictors[6]. The complex model approach offered the advantage of potentially capturing more predictive relationships and reducing bias if truly important predictors had not been identified (due to the underpowered analysis)[4]. However, given our small sample size (n = 40 at baseline, reduced to n = 15 by T4), incorporating numerous predictors risked overfitting the imputation models, potentially introducing noise rather than meaningful data[5]. Conversely, the simplified model using only statistically significant predictors (baseline awareness of exercise recommendations and T2 implementation intentions) risked omitting relevant but underpowered predictors, potentially leading to less efficient imputations[7].

We conducted pilot imputations using both approaches and compared their convergence properties and stability. The simplified approach demonstrated superior convergence characteristics and reduced computational complexity while maintaining imputation quality. Based on these considerations and our relatively small sample size, we selected the more parsimonious simplified model to avoid overfitting[4]. This decision prioritised stability and reduced variance in the imputation models while still incorporating the most strongly predictive variables.

S1.3.2. Distribution analysis

Before selecting an imputation method, we examined the distributional properties of all outcome variables. Visual inspections using histograms, Q-Q plots, and boxplots were conducted, along with the calculation of Shapiro-Wilk statistics. The distribution analyses revealed significant departures from normality for most of our outcome measures. Specifically, most subscales exhibited non-normal distributions with varying skewness and kurtosis across measurement timepoints. These distribution characteristics informed both our imputation approach and subsequent modelling strategy.

S1.3.3. Multiple imputation

Multiple imputation was performed using predictive mean matching (PMM) as the imputation method. PMM was selected as it preserves the distributional characteristics of the original data while accommodating non-normality, and maintains the original scale of the variables[8].

Following recommendations for datasets with substantial missingness, defined as missingness rates exceeding 40.0%[9], we created 20 imputed datasets to ensure adequate statistical efficiency and maintain power[10]. Separate imputation models were created for ProQOL, PSC, and TDF scales to account for their different measurement patterns and timepoints. Each scale was imputed separately using time, baseline awareness of exercise recommendations, and T2 implementation intentions as predictors in the imputation models. Random intercepts by participant were included to account for individual differences across repeated measurements.

Imputation convergence was assessed using Gelman-Rubin statistics, with 50 iterations sufficient to achieve convergence across all imputed variables (all Gelman-Rubin statistics < 1.05). Diagnostic plots confirmed appropriate mixing of the imputation chains and reasonable imputed values that preserved the distributional characteristics of the observed data.

S1.3.4. Model selection

We systematically evaluated multiple modelling approaches for each subscale, including beta generalised linear mixed models (GLMMs) with three different link functions (logit, probit, and complementary log-log), two random effects structures (random intercepts only vs. random slopes), and three correlation structures (none, autoregressive AR1, and compound symmetry)[11]. Model comparisons using Akaike Information Criterion (AIC)[12] identified beta GLMMs as consistently providing the best fit across all subscales. Specifically, beta GLMMs with complementary log-log link functions demonstrated superior fit for 9 of the 13 subscales, with beta GLMMs using logit or probit links performing best for the remaining subscales. Beta GLMMs were particularly appropriate for all outcome measures due to their ability to handle bounded responses and accommodate non-normal distributions with varying skewness.

S1.3.5. Statistical modelling

For consistency and interpretability, we standardised our approach by applying beta GLMMs with complementary log-log links to all subscales. This decision was made to facilitate comparisons across outcomes while maintaining the appropriate fit properties of beta distributions for our outcome measures. Each model included participant-specific random intercepts, with time as a factor variable, and T1 awareness of exercise guidelines and T2 implementation intentions as fixed effects. The ProQOL and PSC subscales were analysed across all four timepoints (T1 to T4), while TDF subscales were analysed either from T2 to T3 or T2 to T4, depending on the specific subscale's data availability.

Results were pooled across the 20 imputed datasets using Rubin's rules[13] to account for both within- and between-imputation variance in parameter estimates and standard errors. For significant findings, post-hoc analyses were conducted to examine pairwise comparisons between timepoints (using Tukey's adjustment for multiple comparisons). Effect sizes were reported as hazard ratios (HRs), which represent the relative change in the hazard of higher outcome scores associated with each predictor: HR = 1.2/0.83 (20.0% change), medium effects around HR = 1.5/0.67 (50.0% change), and large effects around HR = 2.0/0.5 (100.0% change)[14].

S1.3.6. Sensitivity analyses

We conducted sensitivity analyses to assess the robustness of our findings by comparing results from complete case analysis to our multiple imputation approach[15](Table S4). Overall agreement between the two methods was high (93.9% agreement across 33 parameters), with an average absolute difference in parameter estimates of 0.09 and an average relative difference of 43.1%. Only two parameters showed disagreement in statistical significance, both related to the Secondary Traumatic Stress subscale, where the complete case analysis identified significant effects for T3 and T4 timepoints (p = .04 for both) that were not significant in the pooled imputation analysis (p = .10 and p = .11, respectively). These discrepancies could reflect either the reduced power in the complete case analysis, or the practical challenges of imputing substantial amounts of missing data. While we implemented methodologically sound imputation procedures, the accuracy of imputed values inevitably becomes more uncertain as the proportion of missing data increases, particularly at later timepoints where observed data are sparse.

Given these considerations, the high overall agreement between methods provides reassurance about the stability of our main findings. We primarily report the multiple imputation results in the main text, as they utilised more of the available data and provided protection against bias if data are missing at random[16].

Table S4. Sensitivity Analysis: Comparison of Multiple Imputation versus Complete Case Analysis Results for Key Model Parameters

Outcome measure	Parameter	CC result	MI result	Agreement
PSC_SC	Time (ref: T1)			
	T2 vs T1	-1.28 (<.001)***	-1.27 (<.001)***	✓
	T3 vs T1	-1.81 (<.001)***	-1.76 (<.001)***	✓
	T4 vs T1	-1.88 (<.001)***	-1.75 (<.001)***	✓
PSC_ATP	Time (ref: T1)			
	T2 vs T1	0.39 (.09)	0.42 (.08)	✓
	T3 vs T1	0.27 (.27)	0.31 (.27)	✓
	T4 vs T1	0.11 (.61)	0.24 (.34)	✓
PSC_NHT	Time (ref: T1)			
	T2 vs T1	0.15 (.61)	0.04 (.89)	✓
	T3 vs T1	-0.49 (.15)	-0.43 (.15)	✓
	T4 vs T1	-0.22 (.55)	-0.18 (.55)	✓
TDF_KNO	Time (ref: T2)			
	T3 vs T2	-0.97 (<.001)***	-0.97 (<.001)***	✓
TDF_SKI	Time (ref: T2)			
	T3 vs T2	-0.99 (<.001)***	-0.98 (<.001)***	✓
TDF_PRO	Time (ref: T2)			
	T3 vs T2	-0.33 (.26)	-0.33 (.26)	✓

	T4 vs T2	0.11 (.61)	-0.16 (.60)	✓
TDF_BELCA	Time (ref: T2)			
	T3 vs T2	-0.13 (.63)	-0.13 (.63)	✓
	T4 vs T2	-0.01 (.97)	-0.01 (.97)	✓
TDF_BELCO	Time (ref: T2)			
	T3 vs T2	0.47 (<.001)***	0.47 (<.001)***	✓
	T4 vs T2	0.34 (.08)	0.34 (.08)	✓
TDF_OPT	Time (ref: T2)			
	T3 vs T2	-0.11 (.72)	-0.05 (.88)	✓
	T4 vs T2	-0.03 (.91)	-0.12 (.71)	✓
TDF_INT	Time (ref: T2)			
	T3 vs T2	0.09 (.63)	0.09 (.63)	✓
	T4 vs T2	-0.15 (.59)	-0.15 (.59)	✓
ProQOL_B	Time (ref: T1)			
	T2 vs T1	0.14 (.31)	0.20 (.20)	✓
	T3 vs T1	0.20 (.18)	0.29 (.14)	✓
	T4 vs T1	0.31 (.07)	0.41 (.06)	✓
ProQOL_C	Time (ref: T1)			
	T2 vs T1	-0.13 (.14)	-0.04 (.76)	✓
	T3 vs T1	-0.09 (.37)	-0.17 (.32)	✓
	T4 vs T1	-0.13 (.24)	-0.18 (.26)	✓
ProQOL_STS	Time (ref: T1)			
	T2 vs T1	0.13 (.44)	0.21 (.25)	✓
	T3 vs T1	0.18 (.04)*	0.33 (.10)	✗
	T4 vs T1	0.15 (.04)*	0.28 (.11)	✗

Note. This sensitivity analysis compared results from Complete Case (CC) analysis versus Multiple Imputation (MI) analysis to assess the robustness of findings to missing data handling approaches. Time points: T1 = baseline; T2 = immediately post-education; T3 = post-implementation with clients; T4 = 12-month follow-up. Scales: PSC = Practitioner Self-Confidence Scale (SC = Self-Confidence, ATP = Attitudes Towards Patients, NHT = Natural History and Treatment of MS); TDF = Theoretical Domains Framework (KNO = Knowledge, SKI = Skills, PRO = Professional Role, BELCA = Beliefs About Capabilities, BELCO = Beliefs About Consequences, OPT = Optimism, INT = Intentions); ProQOL = Professional Quality of Life Scale (B = Burnout, C = Compassion Satisfaction, STS = Secondary Traumatic Stress). Agreement: ✓ = both analyses reach same significance conclusion; ✗ = disagreement in statistical significance. Significance levels: $^{*}p < .05$, $^{**}p < .01$, $^{***}p < .001$.

Supplementary Materials S2. Supplementary results

Table S5. Participant demographic and professional characteristics at each time point

Characteristic	Result at each time point			
	**T1**<br>N = 40	**T2**<br>N = 23	**T3**<br>N = 16	**T4**<br>N=15
**Age**	35.4 ± 9.8	36.4 ± 10.2	36.4 ± 10.4	36.6 ± 10.8
**Sex**				
Female	31 (77.5%)	18 (78.3%)	12 (75.0%)	11 (73.3%)
Male	9 (22.5%)	5 (21.7%)	4 (25.0%)	4 (26.7%)
**Region**				
NSW	5 (12.5%)	2 (8.7%)	1 (6.2%)	1 (6.6%)
QLD	13 (32.5%)	10 (43.5%)	7 (43.8%)	7 (46.7%)
SA	1 (2.5%)	1 (4.3%)	1 (6.2%)	1 (6.6%)
TAS	0 (0.0%)	0 (0.0%)	0 (0.0%)	0 (0.0%)
VIC	8 (20.0%)	4 (17.4%)	3 (18.8%)	2 (13.3%)
WA	11 (27.5%)	6 (26.1%)	4 (25.0%)	4 (26.8%)
NT	1 (2.5%)	0 (0.0%)	0 (0.0%)	0 (0.0%)
ACT	1 (2.5%)	0 (0.0%)	0 (0.0%)	0 (0.0%)
**Clinical role**				
Physiotherapist	20 (50.0%)	14 (60.9%)	12 (75.0%)	11 (73.3%)
Exercise physiologist	20 (50.0%)	9 (39.1%)	4 (25.0%)	4 (26.7%)
**Primary area of work**				
Private clinic	32 (80.0%)	19 (82.7%)	13 (81.4%)	13 (86.6%)
Not for profit	3 (7.5%)	1 (4.3%)	1 (6.2%)	1 (6.7%)
State health authority	2 (5.0%)	2 (8.7%)	1 (6.2%)	0 (0.0%)
Other	3 (7.5%)	1 (4.3%)	1 (6.2%)	1 (6.7%)
**Caseload, neurological**				
0	0 (0.0%)	0 (0.0%)	0 (0.0%)	0 (0.0%)
1 - 50%	19 (47.5%)	12 (52.2%)	6 (37.5%)	5 (33.3%)
51 - 100%	21 (52.5%)	11 (47.8%)	10 (62.5%)	10 (66.7%)
**Caseload, MS**				
0	5 (12.5%)	4 (17.4%)	1 (6.3%)	1 (6.7%)
1 - 50%	31 (77.5%)	16 (69.6%)	12 (75.0%)	11 (73.3%)
51 - 100%	4 (10.0%)	3 (13.0%)	3 (18.7%)	3 (20.0%)

Note. Values are presented as M ± SD for continuous variables and n (%) for categorical variables.

Journal of Clinical Medicine

MDPI

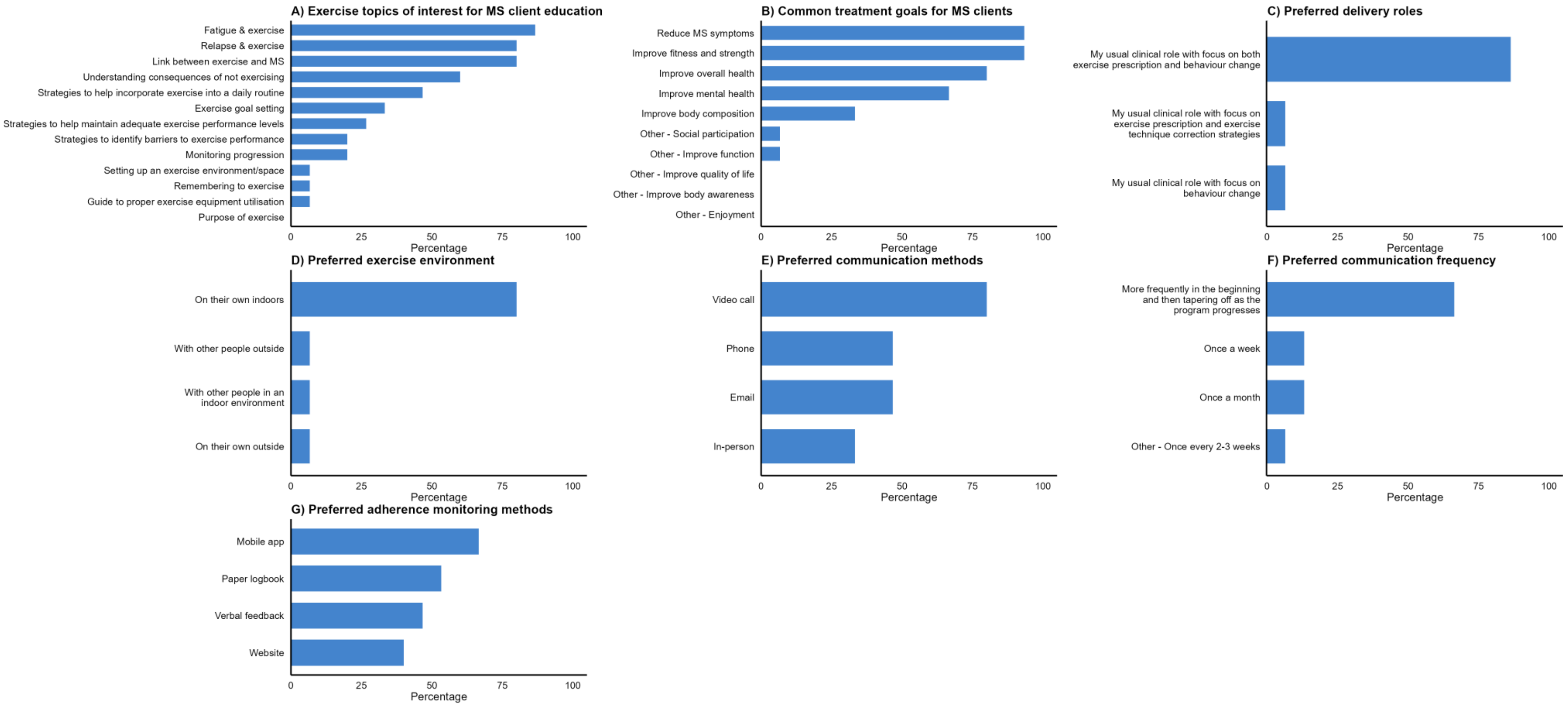

Figure S1. Participants baseline preferences for an education programme to support remote exercise delivery to persons with MS. (**a**) Exercise topics of interest for MS client education. (**b**) Common treatment goals for MS clients. (**c**) Preferred delivery roles. (**d**) Preferred exercise environment. (**e**) Preferred communication methods. (**f**) Preferred communication frequency. (**g**) Preferred adherence monitoring methods.

Table S6. Generalised Linear Mixed Model Results for Practitioner Self-Confidence, Theoretical Domains Framework, and Professional Quality of Life Following the BASE-HCP Programme (N = 40)

Outcome Measure	Parameter	Result (β (SE), z, p)	Effect size (HR (% change) [95% CI])	Predictor context
PSC_SC	Intercept	-0.01 (0.27) -0.06 .96	0.99 (-1.47%) [0.58, 1.67]	
	Time (ref: T1)			
	T2 v T1	-1.27 (0.24) -5.28 < .001	0.28 (-71.97%) [0.17, 0.45]***	
	T3 v T1	-1.76 (0.25) -6.99 < .001	0.17 (-82.74%) [0.11, 0.28]***	
	T4 v T1	-1.75 (0.31) -5.67 < .001	0.17 (-82.58%) [0.09, 0.32]***	
	T1 awareness (ref: no)	-0.52 (0.22) -2.34 .02	0.60 (-40.31%) [0.39, 0.92]*	No: M = 6.3, Yes: M = 4.9*
	T2 intentions	-<0.01 (<0.01) -0.45 .65	1.00 (-0.15%) [0.99, 1.01]	r = -.03
PSC_ATP	Intercept	-1.82 (0.38) -4.76 < .001	0.16 (-83.73%) [0.08, 0.35]	
	Time (ref: T1)			
	T2 v T1	0.41 (0.23) 1.77 .08	1.50 (+49.94%) [0.95, 2.36]	
	T3 v T1	0.31 (0.27) 1.13 .27	1.36 (+36.22%) [0.79, 2.36]	
	T4 v T1	0.17 (0.34) 0.51 .62	1.19 (+18.59%) [0.60, 2.35]	
	T1 awareness (ref: no)	0.46 (0.30) 1.53 .13	1.58 (+58.09%) [0.88, 2.85]	No: M = 5.4, Yes: M = 5.8
	T2 intentions	<0.01 (<0.01) 0.36 .72	1.00 (+0.16%) [0.99, 1.01]	r = .08
PSC_NHT	Intercept	-1.42 (0.34) -4.12 < .001	0.24 (-75.79%) [0.12, 0.48]	

	Time (ref: T1)		1.00 (-0.15%) [0.56, 1.78]	
	T2 v T1	<-0.01 (0.29) -0.01 .100		
	T3 v T1	-0.43 (0.29) -1.46 .15	0.65 (-34.83%) [0.36, 1.17]	
	T4 v T1	-0.18 (0.30) -0.60 .55	0.83 (-16.55%) [0.46, 1.52]	
	T1 awareness (ref: no)	-0.46 (0.25) -1.82 .07	0.63 (-36.75%) [0.39, 1.04]	No: M = 3.6, Yes: M = 3.1
	T2 intentions	<0.01 (<0.01) 1.22 .22	1.00 (+0.41%) [1.00, 1.01]	r = .09
TDF_KNO	Intercept	-0.25 (0.32) -0.77 .44	0.78 (-21.84%) [0.42, 1.47]	
	Time (ref: T2)			
	T3 v T2	-0.97 (0.29) -3.39 < .001	0.38 (-62.14%) [0.21, 0.67]***	
	T1 awareness (ref: no)	-0.48 (0.27) -1.82 .07	0.62 (-38.34%) [0.37, 1.04]	No: M = 1.6, Yes: M = 1.3
	T2 intentions	-0.01 (<0.01) -1.99 .05	0.99 (-0.83%) [0.98, 1.00]	r = -.22
TDF_SKI	Intercept	-0.25 (0.32) -0.78 .43	0.78 (-22.17%) [0.42, 1.46]	
	Time (ref: T2)			
	T3 v T2	-0.98 (0.28) -3.49 < .001	0.37 (-62.65%) [0.21, 0.65]***	
	T1 awareness (ref: no)	-0.89 (0.31) -2.93 .004	0.41 (-59.12%) [0.22, 0.75]**	No: M = 1.7, Yes: M = 1.3**
	T2 intentions	<-0.01 (<0.01) -0.62 .54	1.00 (-0.24%) [0.99, 1.01]	r = -.08
TDF_PRO	Intercept	-0.85 (0.33) -2.58 .01	0.43 (-57.23%) [0.22, 0.82]	
	Time (ref: T2)			

	T3 v T2	-0.33 (0.29) -1.13 .26	0.72 (-27.90%) [0.41, 1.28]	
	T4 v T2	-0.16 (0.30) -0.52 .60	0.86 (-14.38%) [0.47, 1.55]	
	T1 awareness (ref: no)	-0.65 (0.31) -2.08 .04	0.52 (-47.63%) [0.28, 0.97]*	No: M = 2.0, Yes: M = 1.6*
	T2 intentions	<-0.01 (<0.01) -1.22 .23	1.00 (-0.46%) [0.99, 1.00]	r = -.13
TDF_BELCA	Intercept	-0.52 (0.35) -1.49 .14	0.59 (-40.64%) [0.30, 1.19]	
	Time (ref: T2)			
	T3 v T2	-0.13 (0.26) -0.49 .63	0.88 (-12.05%) [0.52, 1.48]	
	T4 v T2	-0.01 (0.27) -0.04 .97	0.99 (-0.99%) [0.58, 1.68]	
	T1 awareness (ref: no)	-0.66 (0.29) -2.26 .03	0.52 (-48.40%) [0.29, 0.92]*	No: M = 2.0, Yes: M = 1.6*
	T2 intentions	<0.01 (<0.01) -0.13 .90	1.00 (-0.05%) [0.99, 1.01]	r < -.01
TDF_BELCO	Intercept	-0.27 (0.21) -1.26 .21	0.77 (-23.36%) [0.50, 1.17]	
	Time (ref: T2)			
	T3 v T2	0.47 (0.15) 3.05 .003	1.60 (+60.13%) [1.18, 2.17]**	
	T4 v T2	0.34 (0.19) 1.80 .08	1.41 (+40.86%) [0.96, 2.06]	
	T1 awareness (ref: no)	0.12 (0.18) 0.67 .51	1.13 (+12.91%) [0.79, 1.62]	No: M = 2.6, Yes: M = 2.7
	T2 intentions	-<0.01 (<0.01) -0.24 .81	1.00 (-0.06%) [0.99, 1.00]	r = -.03
TDF_OPT	Intercept	-0.24 (0.29) -0.82 .41	0.79 (-21.29%) [0.44, 1.40]	
	Time (ref: T2)		0.96 (-4.43%) [0.53, 1.71]	

	T3 v T2	-0.05 (0.29) -0.16 .88		
	T4 v T2	-0.12 (0.32) -0.37 .71	0.89 (-11.08%) [0.47, 1.68]	
	T1 awareness (ref: no)	-0.15 (0.23) -0.64 .52	0.86 (-13.97%) [0.54, 1.37]	No: M = 2.1, Yes: M = 2.0
	T2 intentions	<0.00 (<0.00) 0.65 .52	1.00 (+0.21%) [1.00, 1.01]	r = .03
TDF_INT	Intercept	-0.86 (0.24) -3.55 < .001	0.42 (-57.76%) [0.26, 0.68]	
	Time (ref: T2)			
	T3 v T2	0.09 (0.19) 0.48 .63	1.10 (+9.79%) [0.75, 1.61]	
	T4 v T2	-0.15 (0.29) -0.54 .59	0.86 (-14.26%) [0.48, 1.53]	
	T1 awareness (ref: no)	-0.16 (0.17) -0.94 .35	0.85 (-14.51%) [0.62, 1.19]	No: M = 67.4, Yes: M = 57.9
	T2 intentions	0.01 (<0.01) 4.92 < .001***	1.01 (+1.46%) [1.01, 1.02]***	r = .54
ProQOL_B	Intercept	-1.08 (0.28) -3.87 < .001	0.34 (-66.18%) [0.19, 0.59]	
	Time (ref: T1)			
	T2 v T1	0.20 (0.16) 1.29 .20	1.22 (+22.16%) [0.90, 1.66]	
	T3 v T1	0.29 (0.19) 1.50 .14	1.34 (+34.03%) [0.91, 1.98]	
	T4 v T1	0.41 (0.21) 1.91 .06	1.51 (+50.58%) [0.98, 2.31]	
	T1 awareness (ref: no)	0.31 (0.22) 1.43 .15	1.36 (+36.41%) [0.89, 2.09]	No: M = 19.3, Yes: M = 21.3
	T2 intentions	<0.01 (<0.01) 1.16 .25	1.00 (+0.38%) [1.00, 1.01]	r = .21
ProQOL_C	Intercept	0.13 (0.22) 0.62 .54	1.14 (+14.33%) [0.75, 1.75]	

	Time (ref: T1)		0.96 (-3.86%) [0.74, 1.24]	
	T2 v T1	-0.04 (0.13) -0.30 .76		
	T3 v T1	-0.17 (0.17) -0.99 .32	0.85 (-15.39%) [0.60, 1.18]	
	T4 v T1	-0.18 (0.16) -1.13 .26	0.84 (-16.45%) [0.61, 1.15]	
	T1 awareness (ref: no)	0.30 (0.18) 1.64 .10	1.35 (+35.34%) [0.94, 1.94]	No: M = 40.4, Yes: M = 43.0
	T2 intentions	<-0.01 (<0.01) -0.47 .64	1.00 (-0.11%) [0.99, 1.00]	r = -.12
ProQOL_STS	Intercept	-1.98 (0.26) -7.72 < .001	0.14 (-86.21%) [0.08, 0.23]	
	Time (ref: T1)			
	T2 v T1	0.21 (0.18) 1.17 .25	1.23 (+23.18%) [0.86, 1.76]	
	T3 v T1	0.33 (0.19) 1.68 .10	1.39 (+38.55%) [0.94, 2.05]	
	T4 v T1	0.28 (0.17) 1.62 .11	1.32 (+32.44%) [0.94, 1.87]	
	T1 awareness (ref: no)	0.73 (0.19) 3.80 < .001	2.07 (+106.99%) [1.42, 3.01]***	No: M = 14.8, Yes: M = 18.9***
	T2 intentions	0.01 (<0.01) 2.47 .02	1.01 (+0.7%) [1.00, 1.01]*	r = 0.29*

Note. T1 = baseline; T2 = immediately post-education; T3 = post-implementation with clients; T4 = 12-month follow-up. PSC = Practitioner Self-Confidence Scale (SC = Self-Confidence, ATP = Attitudes Towards Patients, NHT = Natural History and Treatment of MS); TDF = Theoretical Domains Framework (KNO = Knowledge, SKI = Skills, PRO = Professional Role, BELCA = Beliefs About Capabilities, BELCO = Beliefs About Consequences, OPT = Optimism, INT = Intentions); ProQOL = Professional Quality of Life Scale (B = Burnout, C = Compassion Satisfaction, STS = Secondary Traumatic Stress).

Results are from Beta generalised linear mixed models with cloglog link function and participant random intercepts. Fixed effects included time (factor), T1 awareness of MS exercise guidelines (T1 awareness; Yes or No), and T2 (post-learning) exercise prescription intentions (T2 intentions; 0-100% scale). Missing data were handled using multiple imputation (m = 20 datasets) with results pooled using Rubin's rules. Effect sizes are expressed as hazard ratios with percentage change and 95% confidence intervals. Predictor context provides interpretive information to understand significant effects: For T1 awareness, group means show average subscale scores for participants without (No) versus with (Yes) prior guideline

knowledge – differences between these means indicate the practical magnitude of awareness effects. For T2 intentions, correlations (*r*) show the strength and direction of association between post-training exercise prescription intentions (0-100% scale) and subscale scores – positive correlations indicate higher intentions relate to higher subscale scores, while negative correlations indicate higher intentions relate to lower subscale scores. Asterisks denote significant effect sizes and/or prediction contexts: $^{*}p < 0.05$, $^{**}p < 0.01$, $^{***}p < 0.001$.

Table S7. Perceived client outcomes (n = 15)

Realist evaluation	Question	Thematic responses	Example quotes
Outcomes	What positive outcomes did your clients experience from engaging with the BASE program?	*Physical improvements:* Increased function, physical activity, exercise adherence, mobility<br><br>*Psychological improvements:* Goal attainment, motivation, self-confidence, body positivity, mental health, self-efficacy, accountability<br><br>*Symptom improvements:* Reduced fatigue, pain, and spasticity<br><br>(n = 15)	"A client was able to reach her goal of completing 3-day hike in New Zealand, and has started planning another trip with her friend for the following year. Another client was able to join a body pump class while completing the BASE program, and although she had a relapse with some MS symptoms and needed medication changes, she was able to re-focus her goal"
Mechanisms	Why do you think they have experienced these positive outcomes?	*Programme features:* Accountability, routine, access to equipment, tracking	"The structure and accountability of the programme lead to great adherence...having the programme set and the regular check-ins and recording requirements kept them moving forward over the program. For one who had the most benefits, it was the cutting back at the start of the walking programme and increasing strength work that helped with pain reduction due to doing too

		progress, programme structure<br><br>*Personal factors:* High self-efficacy<br><br>*Support elements:* Good coaches<br><br>(n = 15)	much and pushing herself too hard before the programme starting, that was beneficial for her.”<br><br>“They could see their progress clearly on the spreadsheet. Progress was gradual and not too hard. Improved self-efficacy over time as they saw their own results. Great educational resources and reading prior to commencing.”
Contexts	Under what circumstances do you think they may not have achieved these positive outcomes?	*Program-related barriers:* Not attending calls, lack of coaching/guidance<br><br>*Personal barriers*: Lack of enjoyment, lack of commitment<br><br>*External barriers*: External time pressures<br><br>(n = 10)	“Active disease progression/relapses; Reduced levels of mobility (all my clients were quite mobile and functioning well); Multiple physical or psychological comorbidities – one of my participants dropped out as she had multiple physical and psychological factors. It was hard to keep her engaged remotely as she did not answer calls/emails.”<br><br>“If something unexpected happened such as illness, death, increased requirements at work meaning that they could not stick to the program. As it was a study

		participants feeling like they were getting "behind." In clinical practice there is no deadline like the end of the study so easier to 'reset.'"

References

1. Smucker, D.R.; Konrad, T.R.; Curtis, P.; Carey, T.S. Practitioner Self-Confidence and Patient Outcomes in Acute Low Back Pain. *Arch. Fam. Med.* **1998**, *7*, 223–228, doi:10.1001/archfami.7.3.223.
2. Huijg, J.M.; Gebhardt, W.A.; Crone, M.R.; Dusseldorp, E.; Presseau, J. Discriminant Content Validity of a Theoretical Domains Framework Questionnaire for Use in Implementation Research. *Implement. Sci.* **2014**, *9*, 11, doi:10.1186/1748-5908-9-11.
3. Little, R.J.A. A Test of Missing Completely at Random for Multivariate Data with Missing Values. *J. Am. Stat. Assoc.* **1988**, *83*, 1198–1202, doi:10.1080/01621459.1988.10478722.
4. Collins, L.M.; Schafer, J.L.; Kam, C.M. A Comparison of Inclusive and Restrictive Strategies in Modern Missing Data Procedures. *Psychol. Methods* **2001**, *6*, 330–351.
5. Faisal, S.; Tutz, G. Multiple Imputation Using Nearest Neighbor Methods. *Inf. Sci.* **2021**, *570*, 500–516, doi:10.1016/j.ins.2021.04.009.
6. Chaput-Langlois, S.; Stickley, Z.L.; Little, T.D.; Rioux, C. Multiple Imputation When Variables Exceed Observations: An Overview of Challenges and Solutions. *Collabra Psychol.* **2024**, *10*, 92993, doi:10.1525/collabra.92993.
7. Rubin, D. Multiple Imputation. In *Flexible imputation of missing data*; CRC Press: Boca Raton, USA., 2018.
8. Little, R. Univariate Missing Data. In *Flexible imputation of missing data*; CRC Press: Boca Raton, USA., 2018; pp. 63–103.
9. Bennett, D.A. How Can I Deal with Missing Data in My Study? *Aust. N. Z. J. Public Health* **2001**, *25*, 464–469.
10. Graham, J.W.; Olchowski, A.E.; Gilreath, T.D. How Many Imputations Are Really Needed? Some Practical Clarifications of Multiple Imputation Theory. *Prev. Sci. Off. J. Soc. Prev. Res.* **2007**, *8*, 206–213, doi:10.1007/s11121-007-0070-9.
11. Ferrari, S.; Cribari-Neto, F. Beta Regression for Modelling Rates and Proportions. *J. Appl. Stat.* **2004**, *31*, 799–815, doi:10.1080/0266476042000214501.
12. Burnham, K.; Anderson, D. Information and Likelihood Theory: A Basis for Model Selection and Inference. In *Model selection and multimodel inference: A practical information-theoretic approach*; Springer-Verlag: New York; pp. 49–97 ISBN 978-0-387-95364-9.
13. Rubin, D. Inference and Missing Data. *Biometrika* **1976**, *63*, 581–592, doi:10.1093/biomet/63.3.581.
14. Cox, D.R. Regression Models and Life-Tables. *J. R. Stat. Soc. Ser. B Methodol.* **1972**, *34*, 187–202, doi:10.1111/j.2517-6161.1972.tb00899.x.
15. Carpenter, J.R.; Kenward, M.G.; Vansteelandt, S. A Comparison of Multiple Imputation and Doubly Robust Estimation for Analyses with Missing Data. *J. R. Stat. Soc. Ser. A Stat. Soc.* **2006**, *169*, 571–584, doi:10.1111/j.1467-985X.2006.00407.x.
16. Sterne, J.A.C.; White, I.R.; Carlin, J.B.; Spratt, M.; Royston, P.; Kenward, M.G.; Wood, A.M.; Carpenter, J.R. Multiple Imputation for Missing Data in Epidemiological and Clinical Research: Potential and Pitfalls. *BMJ* **2009**, *338*, b2393, doi:10.1136/bmj.b2393.
